# Concise syntheses of natural diarylheptanoids containing a 1,4-pentadiene unit

**DOI:** 10.1007/s13659-025-00517-8

**Published:** 2025-05-13

**Authors:** Guang Tao, Xin-Yue Hu, Hong-Xing Liu, Xing-Ren Li, Li-Dong Shao, Gang Xu

**Affiliations:** 1 Yunnan Key Laboratory of Southern Medicinal Utilization, School of Chinese Materia Medica, Yunnan University of Chinese Medicine, Kunming, 650500 China; 2 State Key Laboratory of Phytochemistry and Natural Medicines, and Yunnan Key Laboratory of Natural Medicinal Chemistry, Kunming Institute of Botany, Chinese Academy of Sciences, Kunming, 650201 China; 3 University of Chinese Academy of Sciences, Beijing, 100049 China

**Keywords:** Diaryheptanoids, Concise synthesis, 1,4-Pentadiene, Macrocyclization

## Abstract

Two concise and efficient synthetic routes were developed for the synthesis of three 1,7-diarylheptanoids (**1**–**3**) containing a 1,4-pentadiene unit, which were originally isolated from *Ottelia acuminata* var. *acuminata*. The first approach focused on the construction of linear diarylheptanoids **1** and **3** featuring a (1*E*,4*E*)-pentadiene moiety, via a Suzuki coupling reaction. The second strategy enabled the synthesis of sixteen-membered macrocyclic ether **2** with a (1*Z*,4*E*)-pentadiene unit. The challenging macrocyclization was successfully accomplished through an Ullmann coupling. Notably, the formation of the *Z*-olefin within the macrocyclic framework was promoted by the inherent ring strain of diarylether-type heptane system, which preferentially stabilizes this particular configuration.

**Graphical Abstract:**

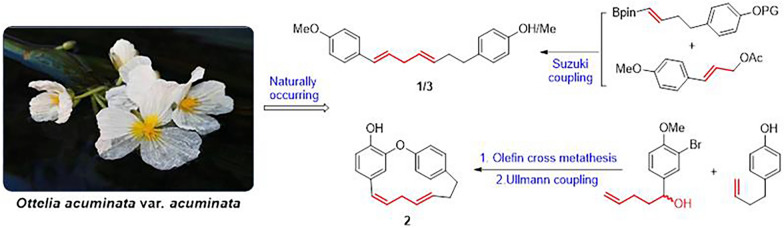

**Supplementary Information:**

The online version contains supplementary material available at 10.1007/s13659-025-00517-8.

## Introduction

**Fig. 1:**
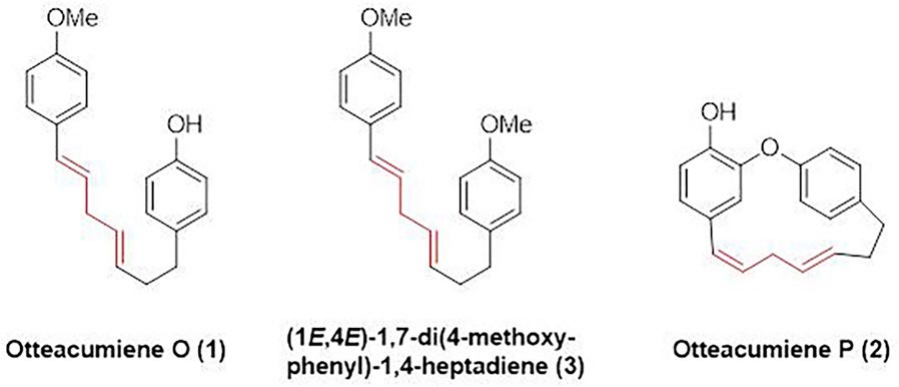
Chemical structures of compounds **1**–**3**

1,7-Diarylheptanes are a class of naturally occurring compounds, primarily isolated from various plants such as *Curcuma longa* (turmeric)*, Zingiber officinale* (ginger)*, Lithospermum erythrorhizon, Aucuba japonica,* and *Betula* species [1]. Structurally, these compounds are categorized into three primary types: linear diarylheptanoids, biarylheptanoids, and diarylether heptanoids. They exhibit a broad spectrum of pharmacological properties, such as antioxidant, anti-inflammatory, antiviral, and anticancer activities [2–4]. Recent phytochemical investigations had led to the isolation of two new diarylheptanoids, otteacumiene O (**1**) and otteacumiene P (**2**)**,** and a known one (1*E*,4*E*)-1,7-di(4-methoxyphenyl)-1,4-heptadiene (**3**) (Fig. 1) from *Ottelia acuminata* var. *acuminata* [5]. Notably, these compounds had shown remarkable *α*-glucosidase inhibitory activity, highlighting their therapeutic potential in regulating postprandial hyperglycemia [6].

Compounds **1**–**3** are characterized by their structural incorporation of a distinctive 1,4-pentadiene moiety, a structural motif frequently observed in biologically active natural products owing to its significant contributions to molecular interactions and conformational flexibility [7]. The 1,4-diene framework is predominantly constructed through a metal-catalyzed coupling reaction, employing transition metal catalysts (e.g., palladium or nickel) to mediate the cross-coupling between allylic alcohols or their corresponding ethers/esters derivatives with various boronic reagents, including boronic acids, boronic esters, or borates [8–10]. Given the significant pharmacological relevance of diarylheptanoids, considerable research efforts have been focused on developing efficient synthetic routes for these compounds. The synthesis of linear diarylheptanoids has been achieved through various well-established methodologies, including Knoevenagel condensation [11], aldol condensation [12], olefin metathesis [13], and Wittig reaction [14]. In contrast, the construction of diarylether heptanoids has been achieved through two main synthetic strategies. The first approach utilizes etherification through Ullmann coupling to form the diarylether linkage, followed by ring closure. The second strategy involves the synthesis of pre-functionalized 1,7-diarylheptane intermediates, which subsequently undergo etherification to afford diarylether heptanoids. Notably, the later approach enables the efficient introduction of the *E*-olefin configuration, thereby meeting the stereochemical requirements of the target compounds. Considering the limited natural abundance of compounds **1**–**3** in *O. acuminata* var. *acuminata*, which precludes comprehensive pharmacological investigation, we have established two efficient synthetic protocols to access these compounds in quantities sufficient for detailed evaluation of their anti-diabetic activity.

## Result and discussion

**Fig. 2:**
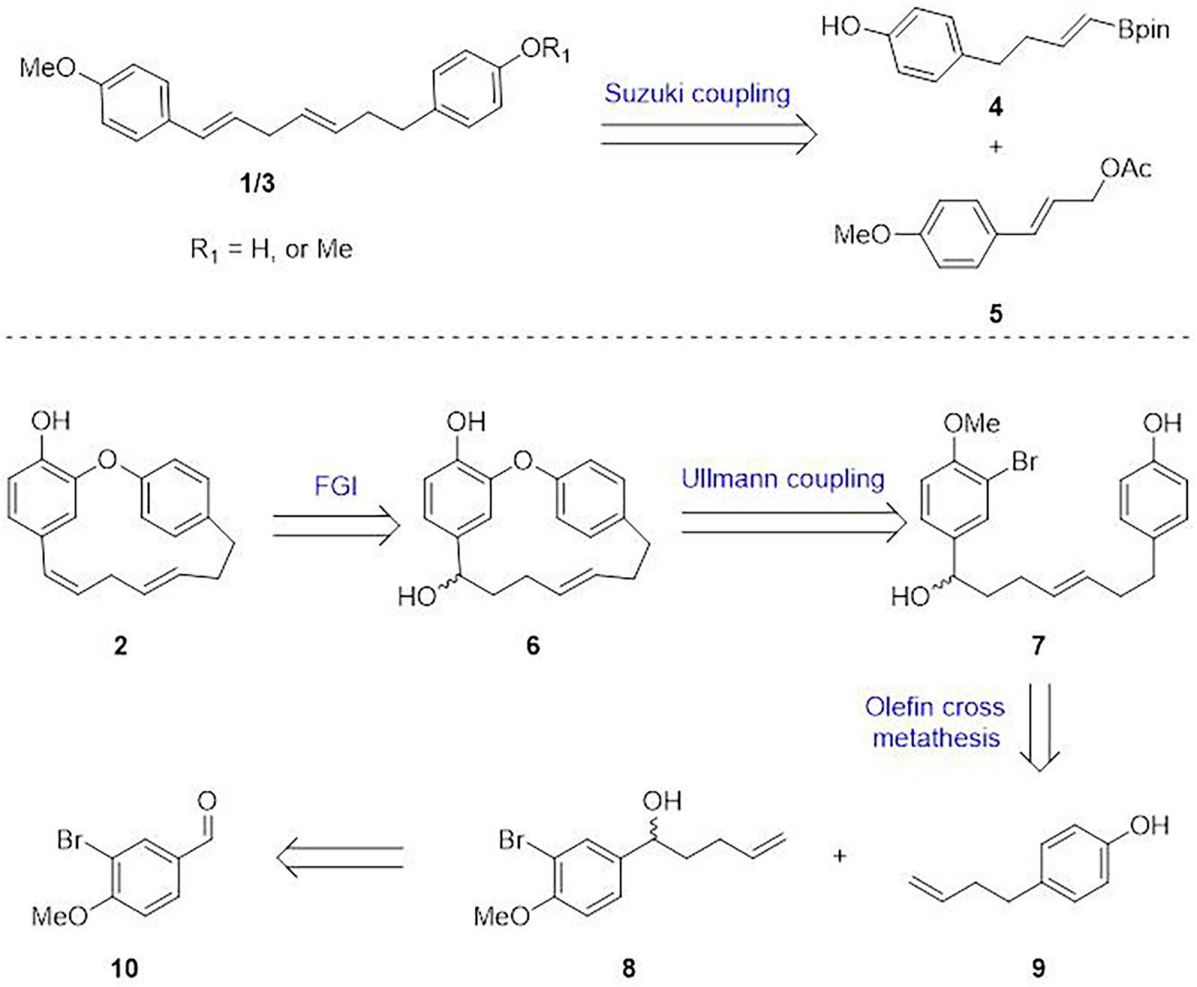
Retrosynthetic analysis of compounds **1**–**3**

As outlined in the retrosynthetic analysis (Fig. 2), we envisioned that both otteacumiene O (**1**) and (1*E*,4*E*)-1,7-di(4-methoxyphenyl)-1,4-heptadiene (**3**) could be efficiently synthesized via a Suzuki coupling reaction between intermediates **4** and **5**, thereby constructing the crucial skipped *E*,*E*-diene structural motif. In parallel, we developed an alternative synthetic route to otteacumiene P (**2**), featuring two pivotal transformations: an olefin cross-metathesis between intermediates **8** and **9**, followed by an intramolecular Ullmann coupling to the macrocyclization (Fig. 2).

**Fig. 3:**
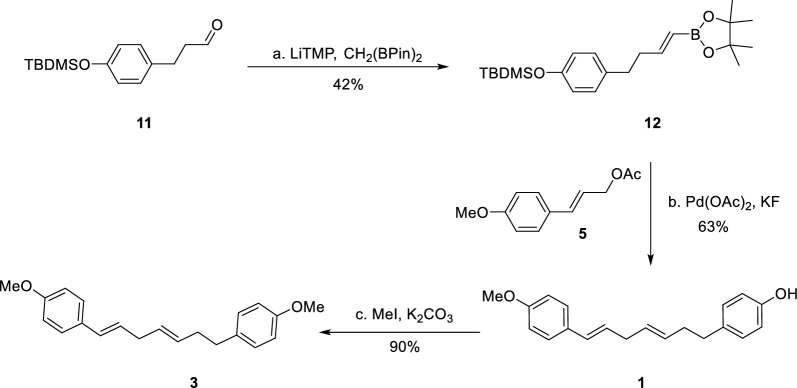
Total synthesis of compounds **1** and **3**. *Reagents and conditions:* **a.** LiTMP (4.0 eq.), CH_2_(BPin)_2_ (3.0 eq.), THF, − 78 °C, 24 h, 42%; **b.** Pd(OAc)_2_ (0.3 eq.), KF (2.5 eq.), EtOH, 70 °C, 24 h, 63%; **c.** MeI (4.0 eq.), K_2_CO_3_ (3.0 eq.), DMF, rt, overnight, 90%. *LiTMP* Lithium tetramethylpiperidide, *THF* tetrahydrofuran

Our synthesis commenced with the preparation of borate ester **12** from a known aldehyde **11** [15], which was previously synthesized from methyl 3-(4-hydroxyphenyl)propionate in 27% overall yield over three steps. Through implementation of Morken's recently developed boron-Wittig protocol [16], employing bis[(pinacolato)boryl]methane as the boron source and our systematic optimizations of reaction parameters, vinyl boronate **12** was obtained in 42% yield with complete stereoselectivity (Fig. 3).

Following literature procedures [17], compound **5** was successfully prepared. With both key intermediates **12** and **5** in hand, a Suzuki coupling reaction was performed. The coupling was efficiently catalyzed by Pd(OAc)₂ in ethanol, affording otteacumiene O (**1**) in 63% yield. Subsequent methylation of compound **1** using methyl iodide provided **3** in excellent yield (90%). This concise synthetic approach enabled efficient access to natural products **1** and** 3** in three steps, achieving an overall yield of 27% from the reported compounds **11** and **5**.

**Fig. 4:**
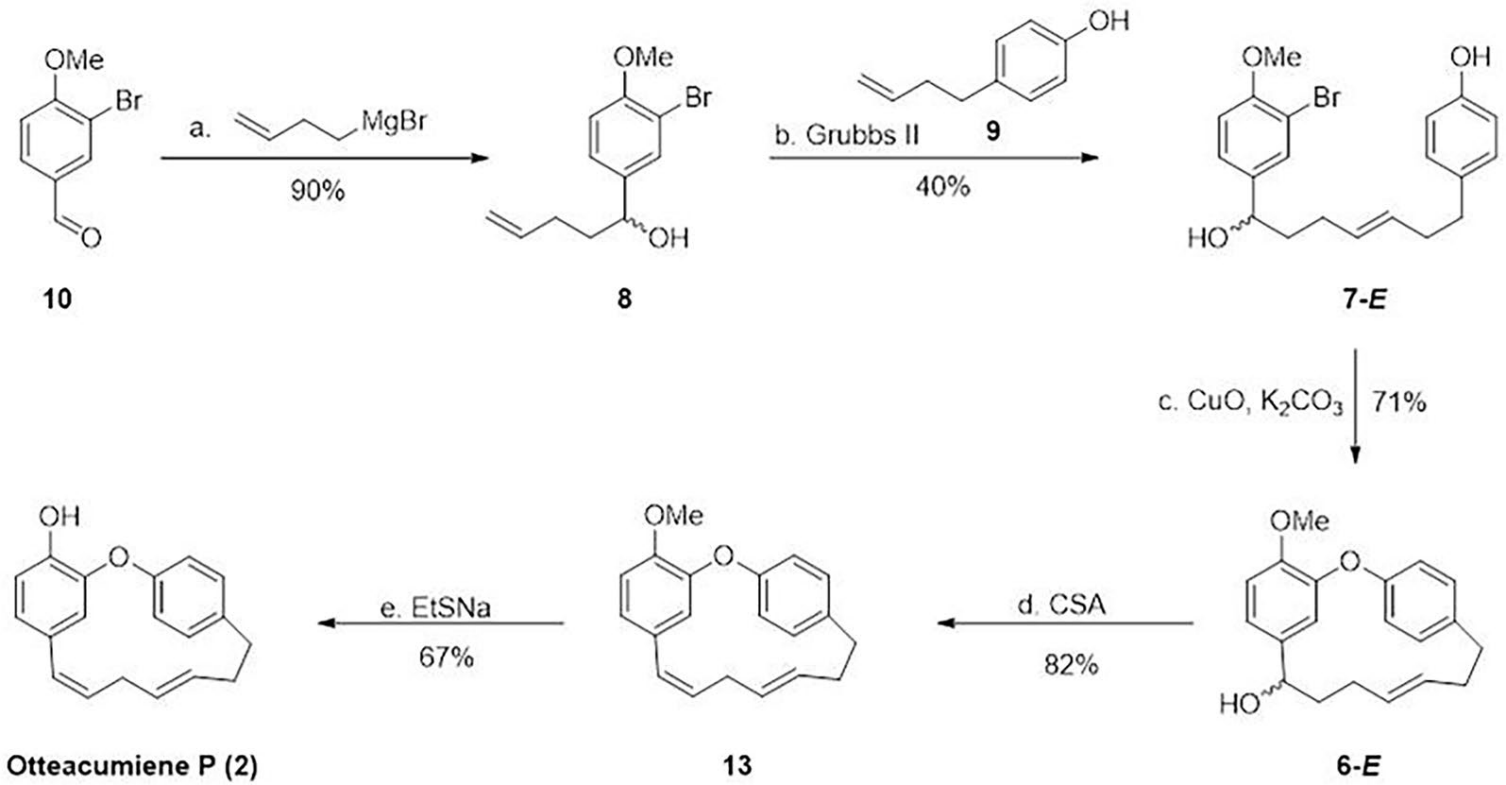
Total synthesis of otteacumiene P (**2**). *Reagents and conditions:* **a.** 3-butenylmagnesium bromide solution (2.5 eq.), THF, 0 °C, 2.0 h 90%; **b** Grubbs-II catalyst (0.1 eq.), DCM, 45 °C, 16 h, 40%; **c.** CuO (5.0 eq.), K_2_CO_3_ (5.0 eq.), pyridine, 150 °C, 71%; **d.** CSA (1.5 eq.), DCM, 60 °C, 2.5 h, 82%; **e.** EtSNa (3.5 eq.), DMF, 150 °C, 3.5 h, 67%. *THF* tetrahydrofuran, *DCM* Dichloromethane, *CSA* Camphor-10-sulfonic acid

Building on the successful syntheses of **1** and **3**, we turned our attention to the more challenging macrocyclic diarylheptanoid **2** (Fig. 4). The synthesis commenced with the preparation of known intermediate **9** from the commercially available 4-hydroxybenzaldehyde through a well-established four steps sequence [18]. For the synthesis of fragment **8**, we initiated the sequence with the condensation of aldehyde **10**, which underwent Grignard addition with 3-butenylmagnesium bromide to afford adduct **8** in 90% yield.

**Fig. 5:**
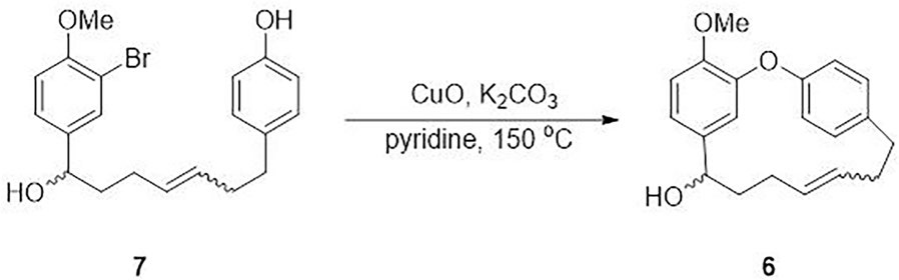
Ullmann coupling of mixture **7**

With both cross-metathesis partners** 8** and **9** readily available, the olefin metathesis reaction was carried out in the presence of Grubbs II catalyst under refluxing DCM, yielding olefin **7-***E* in 40% yield. Notably, the metathesis reaction produced a structurally analogous and co-eluting impurity with similar polarity, which complicated the determination of the *E*/*Z* isomer ratio. To circumvent this issue, we directly subjected the crude cross-metathesis product **7** to an Ullmann coupling reaction (Fig. 5). Subsequent purification by PTLC yielded mixture **6**, which contained only the desired *E*/*Z* isomers. Detailed ^1^H NMR analysis of mixture **6** (see Supporting Information) revealed an *E*/*Z* ratio of 5:1.

The *E*-isomer of **7** was subsequently isolated via semi-prep HPLC employing a chiral stationary phase. The key macrocyclization was then achieved through intramolecular Ullmann coupling of **7-***E* using CuO and K₂CO₃ in refluxing pyridine, providing macrocyclic intermediate **6-***E* in 71% yield. At this stage, we envisioned that the requisite *Z*-alkene could be installed through stereospecific dehydration of **6**-***E***. Indeed, treatment of **6-***E* with camphorsulfonic acid (CSA) in toluene at 60 °C directly afforded compound **13** in 82% yield. The final transformation involved demethylation of **13** using sodium ethanethiolate (EtSNa) in refluxing DMF, which cleanly delivered the target otteacumiene P (**2**) in 67% yield. The ^1^H and ^13^C NMR spectroscopic data of our synthetic **2** were in complete agreement with those of natural** 2**. This synthesis of otteacumiene P (**2**) was successful accomplished in five steps with an overall yield of 14%, representing an efficient and practical route to this biologically relevant natural product.

## Conclusions

In summary, we have successfully achieved the first total synthesis of naturally occurring 1,7-diarylheptanes **1**–**3**. Our synthetic strategy enabled efficient access to linear diarylheptanoids **1** and **3** in three steps with 27% overall yield, while the more structurally complex macrocyclic analogue **2** was synthesized in five steps with 14% overall yield. The synthetic approaches employ an intramolecular Suzuki coupling and an Ullmann macrocyclization to efficiently construct two classes of diarylheptanoids, featuring (1*E*,4*E*)-pentadiene and (1*Z*,4*E*)-pentadiene units, respectively. This efficient synthetic route not only provides sufficient quantities of **1**–**3** for comprehensive biological evaluation but also establishes a versatile platform for the preparation of structural analogues to facilitate detailed structure—activity relationship studies.

## Experimental procedures

### General experimental procedures

Unless otherwise mentioned, all reactions were carried out under an argon atmosphere under anhydrous conditions, and all reagents were purchased from commercial suppliers without further purification. NMR spectra were recorded on Bruker ARX600, and calibrated using residual undeuterated solvent as an internal reference (CDCl_3_, *δ*_H_ 7.26 ppm ^1^H NMR, *δ*_C_ 77.0 ppm ^13^C NMR; Acetone-*d*_6_, *δ*_H_ 2.05 ppm ^1^H NMR, *δ*_C_ 206.3 ppm ^13^C NMR). The following abbreviations were used to explain the multiplicities: s = singlet, d = doublet, t = triplet, q = quartet, b = broad, m = multiplet. High-resolution mass spectra (HRMS) were recorded on a Bruker Apex IV FTMS mass spectrometer using ESI (electrospray ionization).

#### (*E*)-tert-Butyldimethyl(4-(4-(4,4,5,5-tetramethyl-1,3,2-dioxaborolan-2-yl)but-3-en-1-yl)phenoxy)silane (12)

To a two-necked 25 mL flask, 2,2,6,6-tetramethylpiperidine (TMP, 350 *µ*L, 2.32 mmol, 4.0 eq.) and *n*-BuLi (1 M solution in hexane, 2.32 mL, 2.32 mmol, 4.0 eq.) were added at 0 °C. The resulting mixture was stirred for 10 min at the same temperature, followed by the dropwise addition of a solution of bis[(pinacolato)boryl]methane (466 mg, 1.74 mmol, 3.0 eq.) in anhydrous THF (0.7 mL). After an additional 10 min of stirring, the reaction mixture was cooled to – 78 °C and stirred for another 10 min. Then, a solution of **11** (153 mg, 0.58 mmol, 1.0 eq.) in THF (0.7 mL) was added dropwise to the mixture. The reaction was stirred for 2 h at –78 °C, after which excess reagents were quenched by the addition of saturated aqueous NH_4_Cl. The reaction mixture was extracted with EtOAc. The combined organic layers were dried over Na_2_SO_4_, filtered, and concentrated under reduced pressure. The residue was purified by silica gel column chromatography (hexane) to afford **11** as a colorless oil (94 mg, 42%). *R*_f_ = 0.5 (SiO_2_, hexane) This obtained material contained trace amounts of unidentified impurities and residual bis[(pinacolato)boryl]methane, as determined by ^1^H NMR analysis. ^1^H NMR (600 MHz, CDCl_3_) *δ* 7.04 – 7.01 (m, 2H), 6.75 – 6.73 (m, 2H), 6.68 (d, *J* = 17.9 Hz, 1H), 5.48 (d, *J* = 17.9 Hz, 1H), 2.66 (dd, *J* = 9.5, 6.6 Hz, 2H), 2.44 (ddd, *J* = 9.5, 5.0, 1.6 Hz, 2H), 1.27 (s, 12H), 0.97 (s, 9H), 0.18 (s, 6H); ^13^C NMR (151 MHz, CDCl_3_) *δ* 153.8, 153.8, 134.6, 129.3, 129.3, 129.3, 120.0, 120.0, 83.2, 83.2, 37.9, 33.9, 25.9, 25.9, 25.9, 24.9, 24.9, 24.9, 24.9, 18.3, − 4.3, − 4.3; HRMS (ESI): *m*/*z* [M + H]^+^ calcd for C_22_H_37_BO_3_Si: 388.2720; found: 388.2719.

#### (1*E*,4*E*)-1-(4-Hydroxyphenyl)-7-(4-methoxyphenyl)-1,4-heptadiene (Otteacumiene O (1))

To a mixture solution of **12** (48 mg, 0.23 mmol, 1.0 eq.), **5** (152 mg, 0.39 mmol, 1.7 eq.) and KF (34 mg, 0.59 mmol, 2.5 eq.) in anhydrous ethanol (5 mL) was added Pd(OAc)_2_ (15.7 mg, 0.07 mmol, 0.30 eq.) under an argon atmosphere. The reaction mixture was stirred for 24 h at 70 °C. After the reaction was confirmed to be complete by TLC monitoring, the mixture was quenched with water (5 mL) and extracted with dichloromethane (DCM, 2 × 10 mL). The combined organic layers were washed with brine, dried over anhydrous MgSO_4_, and filtered. The organic solvent was completely removed by rotary evaporation. The solid residue was purified by column chromatography (DCM: EtOAc /100:1) to afford otteacumiene O (**1**) (43 mg, 63%) as a yellow oil. *R*_f_ = 0.45 (SiO_2_, DCM: EtOAc /100:1); ^1^H NMR (600 MHz, Acetone-*d*_6_) *δ* 7.31 (d, *J* = 8.7 Hz, 2H), 7.03 (d, *J* = 8.2 Hz, 2H), 6.86 (d, *J* = 8.6 Hz, 2H), 6.74 (d, *J* = 8.4 Hz, 2H), 6.31 (d, *J* = 15.9 Hz, 1H), 6.09 (dt, *J* = 15.8, 6.6 Hz, 1H), 5.59 – 5.45 (m, 2H), 3.78 (s, 3H), 2.84 (m, 2H), 2.59 (dd, *J* = 8.7, 6.7 Hz, 2H), 2.32 – 2.25 (m, 2H); ^13^C NMR (151 MHz, Acetone-*d*_6_) *δ* 159.9, 156.4, 133.5, 131.7, 131.3, 130.6, 130.2, 130.2, 129.4, 127.9, 127.4, 127.4, 115.9, 115.8, 114.7, 114.7, 55.5, 36.5, 35.8, 35.6; HRMS (EI): *m*/*z* [M]^+^ calcd. for C_12_H_14_O_3_: 294.1614; found: 294.1611.

#### (1*E*,4*E*)-1,7-Di(4-methoxyphenyl)-1,4-heptadiene (3)

To a stirred solution of **1** (30 mg, 0.1 mol, 1.0 eq.) and iodomethane (26 *µ*L, 0.4 mmol, 4.0 eq.) in DMF (1 mL) was added K_2_CO_3_ (42 mg, 0.3 mmol, 3.0 eq.). The resultant suspension was heated at 60 °C for 4 h, then allowed to cool to room temperature and stirred overnight. The reaction mixture was filtered, and the solid residue was washed with EtOAc (10 mL). The combined filtrates were then washed with brine (3 × 10 mL). The organic layer was dried over anhydrous MgSO_4_, filtered, and concentrated under reduced pressure. The crude product was purified by flash chromatography on silica gel (hexane: EtOAc/20:1) to give (1*E*,4*E*)-1,7-di(4-methoxyphenyl)-1,4-heptadiene (**3**) (28 mg, 0.9 mmol, 90%) as white solid. *R*_f_ = 0.70 (SiO_2_, PE: EtOAc /5:1); ^1^H NMR (600 MHz, CDCl_3_) *δ* 7.27 (d, *J* = 8.6 Hz, 2H), 7.10 (d, *J* = 8.6 Hz, 2H), 6.84 (d, *J* = 7.4 Hz, 2H), 6.82 (d, *J* = 7.1 Hz, 2H), 6.28 (dt,* J* = 15.7, 1.6 Hz, 1H), 6.05 (dt, *J* = 15.8, 6.6 Hz, 1H), 5.56 – 5.51 (m, 1H), 5.51 – 5.47 (m, 1H), 3.80 (s, 3H), 3.78 (s, 3H), 2.88 – 2.85 (m, 2H), 2.63 (dd, *J* = 8.8, 6.8 Hz, 2H), 2.33 – 2.30 (m, 2H); ^13^C NMR (151 MHz, CDCl_3_) *δ* 158.7, 158.7, 157.7, 157.7, 134.2, 130.8, 129.6, 129.4, 128.6, 127.1, 127.0, 113.9, 113.9, 113.7, 113.7, 55.3, 55.2, 35.9, 35.0, 34.7; HRMS (EI): *m*/*z* [M]^+^ calcd. for C_21_H_24_O_2_: 308.1771; found: 308.1771.

#### 1-(3-Bromo-4-methoxyphenyl)pent-4-en-1-ol (8)

To a solution of **10** (1291 mg, 6.03 mmol, 1.0 eq.) in THF (30 mL), 3-butenylmagnesium bromide solution (1 M, 15 mL, 15.0 mmol, 2.5 eq.) was added dropwise at 0 °C. The reaction was stirred at the same temperature until the starting aldehyde disappeared based on TLC analysis. The mixture was quenched by pouring it into saturated aqueous NH_4_Cl (5 mL) and extracted with EtOAc (20 mL × 3). The organic extracts were washed with brine, dried over MgSO_4_, and then concentrated *in vacuo*. Silica gel column chromatography of the residue (hexane: EtOAc/10:1) gave **8** (1467 mg, 90%) as a white solid. *R*_f_ = 0.35 (SiO_2_, hexane: EtOAc/10:1); ^1^H NMR (600 MHz, CDCl_3_) *δ* 7.21 (d, *J* = 8.3 Hz, 2H), 6.81 (d, *J* = 8.2 Hz, 2H), 5.83 – 5.75 (m, 1H), 5.16 – 5.11 (m, 2H), 4.67 (t, *J* = 6.6 Hz, 1H), 2.49 (t, *J* = 6.9 Hz, 2H), 0.98 (s, 9H), 0.19 (s, 6H); ^13^C NMR (151 MHz, CDCl_3_) *δ* 155.2, 136.8, 134.8, 127.1, 120.1, 118.3, 73.2, 43.9, 25.8, 25.8, 25.8, 18.3, − 4.3, − 4.3; HRMS (ESI): *m*/*z* [M + Na]^+^ calcd for C_12_H_15_BrO_2_: 293.0148; found: 293.0154.

#### (4*E*)-1-Hydroxy-1-(3-bromo-4-methoxyphenyl)-7-(4-hydroxyphenyl)-4-heptene (7)

A solution of **8** (74 mg, 0.28 mmol, 1.0 eq.) and **9** (76 mg, 0.28 mmol, 1.0 eq.) in DCM (5 mL) was degassed for 30 min under a flow of argon. The reaction mixture was heated at reflux using a glycerol bath. Then, Grubbs-II catalyst (10 mol %, 24 mg, 0.028 mmol, 10% eq.) was added in one portion. The reaction mixture was stirred at reflux for 16 h. Upon completion, the solvent was removed and the crude was purified on a flash column (hexanes:acetone/15:1) affording **7** (35 mg, 32%) as a solid. *R*_f_ = 0.2 (SiO_2_, hexanes:acetone/15:1); ^1^H NMR (600 MHz, CDCl_3_) *δ* 7.12 (dd, *J* = 8.3, 2.2 Hz, 1H), 7.07 (dd, *J* = 8.2, 2.5 Hz, 1H), 7.01 (dd, *J* = 8.2, 2.2 Hz, 1H), 6.94 (ddd, *J* = 7.7, 4.9, 2.3 Hz, 2H), 6.88 (d, *J* = 8.2 Hz, 1H), 5.43 (d, *J* = 2.1 Hz, 1H), 5.12 (ddd, *J* = 15.0, 9.1, 5.6 Hz, 1H), 4.95 (dt, *J* = 15.3, 5.9 Hz, 1H), 4.59 (dd, *J* = 6.8, 4.4 Hz, 1H), 3.97 (s, 3H), 2.93–2.88 (m, 1H), 2.70–2.64 (m, 1H), 2.42 (dd, *J* = 13.1, 5.8 Hz, 1H), 2.35 (dd, *J* = 8.8, 6.0 Hz, 1H), 2.10 (dt, *J* = 13.9, 9.6, 4.7 Hz, 2H), 1.83–1.76 (m, 2H); ^13^C NMR (151 MHz, CDCl_3_) *δ* 155.4, 151.3, 147.6, 139.4, 136.7, 132.0, 131.6, 130.4, 129.2, 123.7, 123.0, 118.0, 115.3, 111.7, 72.2, 56.3, 38.8, 35.6, 34.8, 27.56; HRMS (ESI): *m*/*z* [M + Na]^+^ calcd for C_20_H_22_O_3_: 333.1461; found: 333.1464.

#### (*E*)-16-Methoxy-2-oxa-1(1,3),3(1,4)-dibenzenacyclo d-ecaphan-6-en-10-ol (6)

To a solution of **7** (71 mg, 0.176 mmol, 1.0 eq.) in anhydrous pyridine (3 mL) was added K_2_CO_3_ (209 mg, 0.878 mmol, 5.0 eq.). The mixture was heated to 90 °C, followed by the addition of CuO (70 mg; 0.878 mmol, 5.0 eq.). After heating for 48 h, the reaction mixture was cooled to room temperature and concentrated under reduced pressure. The residue was dissolved in EtOAc and neutralized by the addition of 10% aqueous NaHSO_3_ solution. The aqueous layer was extracted with EtOAc, and the combined organic layers were washed with 10% aqueous NaHSO_3_ solution, water, and brine, then dried over Na_2_SO_4_ and concentrated under reduced pressure. The crude residue was purified by column chromatography on silica gel (pentane: EtOAc/10:1) to provide **6** (39 mg, 71%) as a yellow oil. *R*_f_ = 0.25 (SiO_2_, pentane: EtOAc/10:1); ^1^H NMR (600 MHz, CDCl_3_) *δ* 7.12 (dd, *J* = 8.3, 2.2 Hz, 1H), 7.07 (dd, *J* = 8.2, 2.5 Hz, 1H), 7.01 (dd, *J* = 8.2, 2.2 Hz, 1H), 6.94 (ddd, *J* = 7.7, 4.9, 2.3 Hz, 2H), 6.88 (d, *J* = 8.2 Hz, 1H), 5.43 (d, *J* = 2.1 Hz, 1H), 5.12 (ddd,* J* = 15.0, 9.1, 5.6 Hz, 1H), 4.95 (dt, *J* = 15.3, 5.9 Hz, 1H), 4.59 (dd, *J* = 6.8, 4.4 Hz, 1H), 3.97 (s, 3H), 2.93 – 2.88 (m, 1H), 2.70 – 2.64 (m, 1H), 2.42 (dd, *J* = 13.1, 5.8 Hz, 1H), 2.35 (dd, *J* = 8.8, 6.0 Hz, 1H), 2.10 (dt, *J* = 13.9, 9.6, 4.7 Hz, 2H), 1.83 – 1.76 (m, 2H); ^13^C NMR (151 MHz, CDCl_3_) *δ* 155.4, 151.3, 147.6, 139.4, 136.7, 132.0, 131.6, 130.4, 129.2, 123.7, 123.0, 118.0, 115.3, 111.7, 72.2, 56.3, 38.8, 35.6, 34.8, 27.6; HRMS (ESI): *m*/*z* [M + Na]^+^ calcd for C_20_H_22_O_3_: 333.1461; found: 333.1464.

#### (6*E*,9*Z*)-16-Methoxy-2-oxa-1(1,3),3(1,4)-dibenzenacyclo decaphane-6,9-diene (13)

A 10 mL 3-necked flask equipped with a magnetic stirring bar, stoppers, and a nitrogen inlet was charged with **6** (35 mg, 0.122 mmol, 1.0 eq.) dissolved in DCM (3 mL). Camphor-10-sulfonic acid (42 mg, 0.183 mmol, 1.5 eq.) was added and the reaction mixture was stirred at ambient temperature for 2.5 h, until TLC showed complete consumption of the alcohol. The reaction was concentrated and purified via flash column chromatography (pentane: EtOAc/20:1) to yield **13** as a white solid. (29 mg, 82%). *R*_f_ = 0.5 (SiO_2_, pentane: EtOAc/20:1);^1^H NMR (600 MHz, CDCl_3_) *δ* 7.17 – 6.98 (m, 4H), 6.88 (d, *J* = 8.2 Hz, 1H), 6.71 (dd, *J* = 8.3, 2.1 Hz, 1H), 6.26 (d, *J* = 11.4 Hz, 1H), 5.78 (d, *J* = 2.1 Hz, 1H), 5.55 (dt, *J* = 11.4, 9.2 Hz, 1H), 5.23 (dt, *J* = 15.3, 7.7, 2.1 Hz, 1H), 4.39 (dt, *J* = 15.4, 5.6, 1H), 3.98 (s, 3H), 2.77 (s, 2H), 2.63 (s, 2H), 2.34–2.08 (m, 2H); ^13^C NMR (CDCl_3_, 151 MHz, 25 °C) *δ* (four carbons signals were not detectable because of a coalescence at rt) 154.1, 150.8, 147.3, 139.7, 131.3, 129.7, 129.6, 129.5, 128.5, 122.5, 115.5, 111.7, 56.2, 35.7, 35.1, 30.4; HRMS (ESI): *m*/*z* [M + Na]^+^ calcd for C_20_H_20_O_2_: 315.1356; found: 315.1355.

#### (6*E*,9*Z*)-2-Oxa-1(1,3),3(1,4)-dibenzenacyclodecaphane-6,9-dien-16-ol (otteacumiene P (2))

To a solution of **13** (31 mg, 0.106 mmol, 1.0 eq.) in DMF (3 mL) was added EtSNa (30 mg, 0.35 mmol, 3.5 eq.). The reaction mixture was refluxed for 3.5 h. After cooling to 0 °C, 5% aqueous HCl solution was added, the aqueous layer was extracted with EtOAc, and the combined organic layers were successively washed with 5% aqueous HCl solution, H_2_O, and brine. The solution was dried over Na_2_SO_4_ and concentrated under vacuum. The residue was purified by column chromatography on silica gel (pentane: EtOAc/8:1), yielding otteacumiene N (**2**) (29 mg, 67%) as a white solid. *R*_f_ = 0.40 (SiO_2_, pentane: EtOAc/8:1); ^1^H NMR (600 MHz, CDCl_3_) *δ* 7.09 (d, *J* = 7.8 Hz, 2H), 7.04 (d, *J* = 8.0 Hz, 2H), 6.90 (d, *J* = 8.1 Hz, 1H), 6.66 (d, *J* = 8.3 Hz, 1H), 6.26 (d, *J* = 11.5 Hz, 1H), 5.71 (d, *J* = 2.0 Hz, 1H), 5.54 (dt, *J* = 11.6, 9.2 Hz, 1H), 5.22 (dt, *J* = 15.4, 7.7, Hz, 1H), 4.41 (dt, *J* = 15.4, 5.6, 1H), 2.93–2.65 (m, 2H), 2.62 (s, 2H), 2.37–2.11 (m, 2H); ^13^C NMR (CDCl_3_, 151 MHz) *δ* (four carbon signals were not detectable because of a coalescence at rt) 153.6, 148.8, 143.7, 140.2, 131.3, 129.9, 129.5, 129.3, 128.2, 123.0, 115.2, 114.9, 35.7, 35.1, 30.3; HRMS (ESI); *m*/*z* [M + H]^+^ calcd for C_19_H_18_O_2_: 279.1380; found: 279.1386.

## Supplementary Information

Additional file 1.

## Data Availability

The datasets used or analyzed during the current study are available from the corresponding author on reasonable request.
