# Correction: Concise syntheses of natural diarylheptanoids containing a 1,4‑pentadiene unit

**DOI:** 10.1007/s13659-025-00525-8

**Published:** 2025-08-18

**Authors:** Guang Tao, Xin‑Yue Hu, Hong‑Xing Liu, Xing‑Ren Li, Li‑Dong Shao, Gang Xu

**Affiliations:** 1 Yunnan Key Laboratory of Southern Medicinal Utilization, School of Chinese Materia Medica, Yunnan University of Chinese Medicine, Kunming, 650500 China; 2 State Key Laboratory of Phytochemistry and Natural Medicines, and Yunnan Key Laboratory of Natural Medicinal Chemistry, Kunming Institute of Botany, Chinese Academy of Sciences, Kunming, 650201 China; 3 University of Chinese Academy of Sciences, Beijing, 100049 China

**Correction: Natural Products and Bioprospecting (2025) 15:32** 10.1007/s13659-025-00517-8

Following publication of the original article [1], the chemical structures of diarylether heptanoids shown in graphical abstract, Figs. 1, 2, 4, and 5 were corrected as given in this correction.

The chemical structure of compound 2 depicted in the original graphical abstract was incorrect. The revised version with the accurate structure is given in this correction.
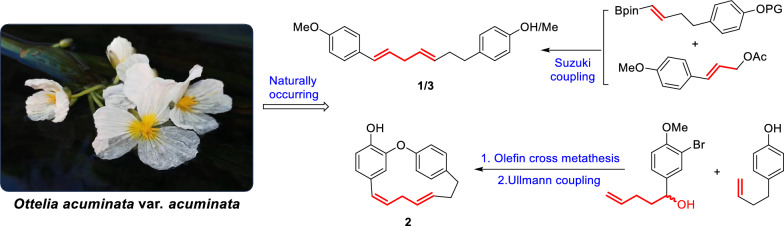


The originally published Fig. 1 contained an incorrect structural representation of otteacumiene P (2). The corrected version (Fig. 1) is provided in this correction.

**Fig. 1:**
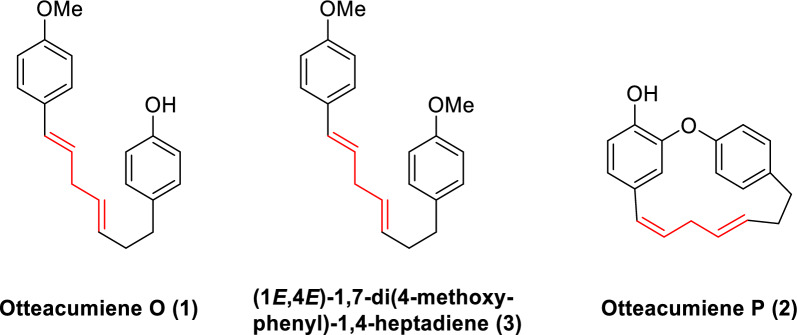
Chemical structures of compounds **1**–**3**

The chemical structures of compounds 2 and 6 in Fig. 2 of the original publication contained inaccuracies. The revised Fig. 2 is presented here (Fig. 2).

**Fig. 2:**
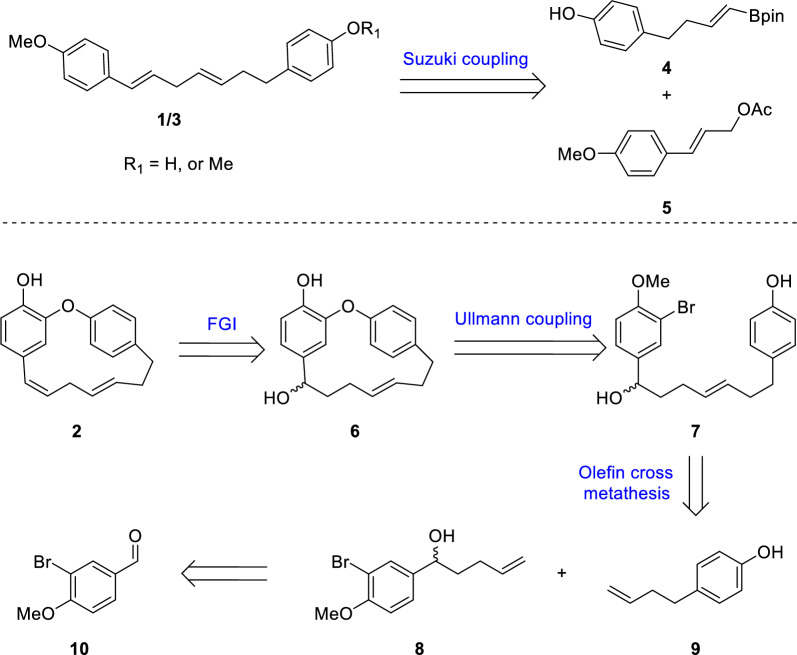
Retrosynthetic analysis of compounds **1**–**3**

The originally published Fig. 4 contained incorrect structural representations of compounds 6-E, 13, and 2. The accurate version of Fig. 4 is presented here (Fig. 4).

**Fig. 4:**
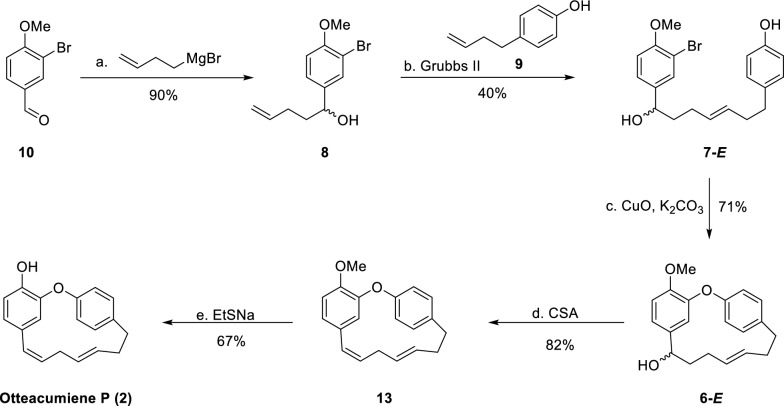
Total synthesis of otteacumiene P (**2**)

The originally published Fig. 5 contained an incorrect structural representation of compound 6. The accurate structure of compound 6 is presented in this erratum (Fig. 5).

**Fig. 5:**
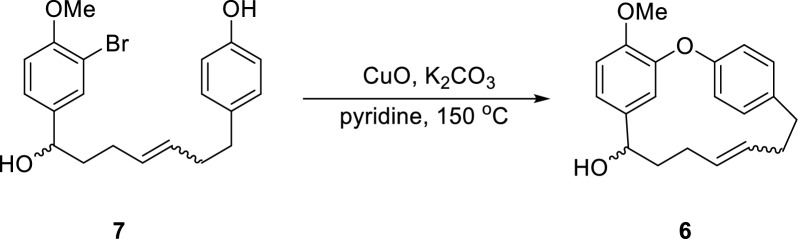
Ullmann coupling of mixture **7**

The original Supporting Information contained numbering errors in both the titles and structures of Figs. S33–37. Corrected versions are presented below.

**Fig. S33:**
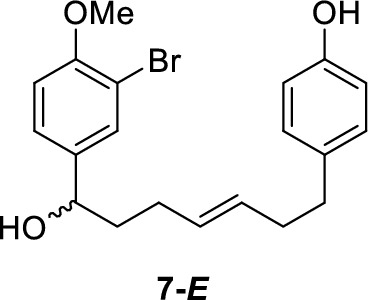
^1^H NMR spectrum of compound **7**-***E*** (CDCl_3_, 600 MHz)

**Fig. S34:**
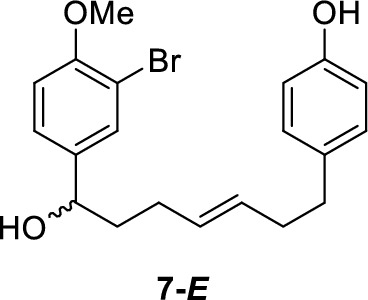
^13^C NMR spectrum of compound **7**-***E*** (CDCl_3_, 150 MHz)

**Fig. S35:**
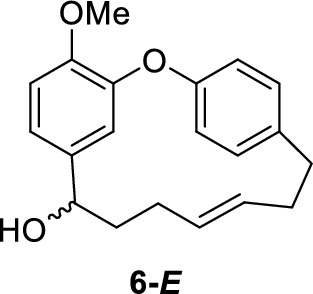
^1^H NMR spectrum of compound **6-*E*** (CDCl_3_, 600 MHz)

**Fig. S36:**
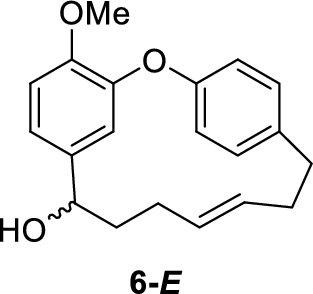
^13^C NMR spectrum of compound **6-*E*** (CDCl_3_, 150 MHz)

**Fig. S37:**
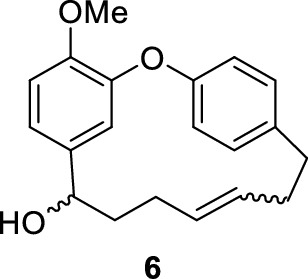
^1^H NMR of compound **6** (provided to determine the ratio of *cis*/*trans* isomers, CDCl_3_, 600 MHz)

The original article [1] has been updated.

## References

[1] Tao G, Hu X-Y, Liu H-X, Li X-R, Shao L-D, Xu S. Concise syntheses of natural diarylheptanoids containing a 1,4-pentadiene unit. Nat Prod Bioprospect. 2025;15:32. 10.1007/s13659-025-00517-8. 40358658 10.1007/s13659-025-00517-8 PMC12075028

