# Supplementary material for: Concise syntheses of natural diarylheptanoids containing a 1,4-pentadiene unit
Source: Nat Prod Bioprospect. 2025 May 13;15(1):32. doi: 10.1007/s13659-025-00517-8 (PMC12075028; doi:10.1007/s13659-025-00517-8)
Supplement: Supplementary file 1 — Additional file 1. [file 13659_2025_517_MOESM1_ESM.docx]

**Supporting information for**

**Concise Syntheses of Natural Diarylheptanoids Containing a 1,4-Pentadiene Unit**

Guang Tao^1^, Xin-Yue Hu^2,3^, Hong-Xing Liu^2^, Xing-Ren Li^2,*^, Li-Dong Shao^1,*^, and Gang Xu^2,^ ^[[1]](#footnote-1)^*

^1^ Yunnan Key Laboratory of Southern Medicinal Utilization, School of Chinese Materia Medica, Yunnan University of Chinese Medicine, Kunming 650500, China

^2^State Key Laboratory of Phytochemistry and Natural Medicines, and Yunnan Key Laboratory of Natural Medicinal Chemistry, Kunming Institute of Botany, Chinese Academy of Sciences, Kunming 650201, China

^3^University of Chinese Academy of Sciences, Beijing 100049, China.

**Table of Contents**

[1. General Information 3](#_Toc192283531)

[2. Synthesis of **11** 3](#_Toc192283532)

[3. Synthesis of **5** 5](#_Toc192283533)

[4. Synthesis of **9** 7](#_Toc192283534)

[5. Synthesis of **10** 9](#_Toc192283535)

[6. NMR spectra copies for all synthetic compounds 11](#_Toc192283536)

[Fig. S1. ^1^H NMR spectrum of compound **17** (CDCl_3_, 600MHz) 11](#_Toc192283537)

[Fig. S2. ^13^C NMR spectrum of compound **17** (CDCl_3_, 150 MHz) 11](#_Toc192283538)

[Fig. S3. ^1^H NMR spectrum of compound **18** (CDCl_3_, 600 MHz) 12](#_Toc192283539)

[Fig. S4. ^13^C NMR spectrum of compound **18** (CDCl_3_, 150 MHz) 12](#_Toc192283540)

[Fig. S5. ^1^H NMR spectrum of compound **11** (CDCl_3_, 600 MHz) 13](#_Toc192283541)

[Fig. S6 .^13^C NMR spectrum of compound **11** (CDCl_3_, 150 MHz) 13](#_Toc192283542)

[Fig. S7. ^1^H NMR spectrum of compound **12** (CDCl_3_, 600 MHz) 14](#_Toc192283543)

[Fig. S8. ^13^C NMR spectrum of compound **12**(CDCl_3_, 150 MHz) 14](#_Toc192283544)

[Fig. S9. ^1^H NMR spectrum of compound **20** (CDCl_3_, 600MHz) 15](#_Toc192283545)

[Fig. S10. ^13^C NMR spectrum of compound **20** (CDCl_3_, 150 MHz) 15](#_Toc192283546)

[Fig. S11. ^1^H NMR spectrum of compound **21** (CDCl_3_, 600 MHz) 16](#_Toc192283547)

[Fig. S12. ^13^C NMR spectrum of compound **21** (CDCl_3_, 150 MHz) 16](#_Toc192283548)

[Fig. S13. ^1^H NMR spectrum of compound **5** (CDCl_3_, 600 MHz) 17](#_Toc192283549)

[Fig. S14. ^13^C NMR spectrum of compound **5** (CDCl_3_, 150 MHz) 17](#_Toc192283550)

[Fig. S15. ^1^H NMR spectrum of compound **1** (Acetone-*d*_6_, 600 MHz) 18](#_Toc192283551)

[Fig. S16. ^13^C NMR spectrum of compound **1** (Acetone-*d*_6_, 150 MHz) 18](#_Toc192283552)

[Fig. S17. ^1^H NMR spectrum of compound **3** (CDCl_3_, 600 MHz) 19](#_Toc192283553)

[Fig. S18 .^13^C NMR spectrum of compound **3** (CDCl_3_, 150 MHz) 19](#_Toc192283554)

[Fig. S19. ^1^H NMR spectrum of compound **23** (CDCl_3_, 600 MHz) 20](#_Toc192283555)

[Fig. S20. ^13^C NMR spectrum of compound **23** (CDCl_3_, 150 MHz) 20](#_Toc192283556)

[Fig. S21. ^1^H NMR spectrum of compound **24** (CDCl_3_, 600 MHz) 21](#_Toc192283557)

[Fig. S22. ^13^C NMR spectrum of compound **24** (CDCl_3_, 150 MHz) 21](#_Toc192283558)

[Fig. S23. ^1^H NMR spectrum of compound **25** (CDCl_3_, 600 MHz) 22](#_Toc192283559)

[Fig. S24. ^13^C NMR spectrum of compound **25** (CDCl_3_, 150 MHz) 22](#_Toc192283560)

[Fig. S25. ^1^H NMR spectrum of compound **9** (CDCl_3_, 600 MHz) 23](#_Toc192283561)

[Fig. S26. ^13^C NMR spectrum of compound **9** (CDCl_3_, 150 MHz) 23](#_Toc192283562)

[Fig. S27. ^1^H NMR spectrum of compound **26** (CDCl_3_, 600 MHz) 24](#_Toc192283563)

[Fig. S28. ^13^C NMR spectrum of compound **26** (CDCl_3_, 150 MHz) 24](#_Toc192283564)

[Fig. S29. ^1^H NMR spectrum of compound **10** (CDCl_3_, 600 MHz) 25](#_Toc192283565)

[Fig. S30. ^13^C NMR spectrum of compound **10** (CDCl_3_, 150 MHz) 25](#_Toc192283566)

[Fig. S31. ^1^H NMR spectrum of compound **8** (CDCl_3_, 600 MHz) 26](#_Toc192283567)

[Fig. S32. ^13^C NMR spectrum of compound **8** (CDCl_3_, 150 MHz) 26](#_Toc192283568)

[Fig. S33.^1^H NMR spectrum of compound **7-*E*** (CDCl_3_, 600 MHz) 27](#_Toc192283569)

[Fig. S34.^13^C NMR spectrum of compound **7-*E*** (CDCl_3_, 150 MHz) 27](#_Toc192283570)

[Fig. S35. ^1^H NMR spectrum of compound **6-*E*** (CDCl_3_, 600 MHz) 28](#_Toc192283571)

[Fig. S36. ^13^C NMR spectrum of compound **6-*E*** (CDCl_3_, 150 MHz) 28](#_Toc192283572)

[Fig. S37. ^1^H NMR of compound **6** (CDCl_3_, 600 MHz) 29](#_Toc192283573)

[Fig. S38. ^1^H NMR spectrum of compound **13** (CDCl_3_, 600 MHz) 29](#_Toc192283574)

[Fig. S39. ^13^C NMR spectrum of compound **13** (CDCl_3_, 150 MHz) 30](#_Toc192283575)

[Fig. S40. ^1^H NMR spectrum of compound **2** (CDCl_3_, 600 MHz) 30](#_Toc192283576)

[Fig. S41. ^13^C NMR spectrum of compound **2** (CDCl_3_, 150 MHz) 31](#_Toc192283577)

[7. HRESIMS spectrum data of important compounds 31](#_Toc192283578)

[Fig. S42. HRESIMS spectrum of otteacumiene O (**1**) 31](#_Toc192283579)

[Fig. S43. HRESIMS spectrum of otteacumiene P (**2**) 32](#_Toc192283580)

[Fig. S44. HRESIMS spectrum of compound **3** 32](#_Toc192283581)

[8. Comparison of NMR data of natural **1** and synthetic **1** 33](#_Toc192283582)

[Fig. S45 Comparison of ^1^H NMR data of natural **1** and synthetic **1** 33](#_Toc192283583)

[Fig. S46 Comparison of ^13^C NMR data of natural **1** and synthetic **1** 34](#_Toc192283584)

[9. Comparison of NMR data of natural **3** and synthetic **3** 34](#_Toc192283585)

[10. Comparison of NMR data of natural **2** and synthetic **2** 35](#_Toc192283586)

[Fig. S47 Comparison of ^1^H NMR data of natural **2** and synthetic **2** 36](#_Toc192283587)

[Fig. S48 Comparison of ^13^C NMR data of natural **2** and synthetic **2** 36](#_Toc192283588)

## 1. General Information

Unless otherwise mentioned, all reactions were carried out under an argon atmosphere under anhydrous conditions and all reagents were purchased from commercial suppliers without further purification. NMR spectra were recorded on Bruker ARX600, and calibrated using residual undeuterated solvent as an internal reference (CDCl_3_, *δ* 7.26 ppm ^1^H NMR, *δ* 77.0 ppm ^13^C NMR; Acetone-*d*_6_, *δ* 2.05 ppm ^1^H NMR, *δ* 206.3 ppm ^13^C NMR;). The following abbreviations were used to explain the multiplicities: s = singlet, d = doublet, t = triplet, q = quartet, b = broad, m = multiplet High-resolution mass spectra (HRMS) were recorded on a Bruker Apex IV FTMS mass spectrometer using ESI (electrospray ionization)

## 2. S**ynthesis of 11**

***Methyl 3-(4-((tert-butyldimethylsilyl)oxy)phenyl)propanoate (17)***. Under nitrogen atmosphere, to a solution of **16** (540 mg, 3 mmol) and imidazole (612 mg, 9 mmol) in DMF (15 mL) was added chloro *tert*-butyldimethylsilane (TBDMSCl, 1.35 g, 9 mmol). The resulting solution was stirred for 24 h at room temperature. The reaction mixture was diluted with hexane/Et_2_O = 1/1 (10 mL), and organic layer was washed three times with H_2_O (10 mL) and once with brine (10 mL), dried over Na_2_SO_4_, filtered, and the filtrate was concentrated to give a colorless liquid, which crystallized on standing to yield as a colorless solid **17** (847 mg, 95%).^1^H NMR (600 MHz, CDCl_3_) *δ* 7.03 (s, 2H), 6.75 (d, *J* = 8.5 Hz, 2H), 3.66 (s, 3H), 2.87 (t, *J* = 7.9 Hz, 2H), 2.61 – 2.55 (m, 2H), 0.97 (s, 9H), 0.18 (s, 6H); ^13^C NMR (150 MHz, CDCl_3_) *δ* 173.6, 154.2, 133.3, 129.3, 129.3, 120.2, 120.2, 51.7, 36.1, 30.3, 25.8, 25.8, 25.8, 18.3, − 4.3, − 4.3; HRMS (EI): *m/z* [M]^+^ calcd for C_16_H_26_O_3_Si: 294.1646; found: 294.1646.

***3-(4-((tert-Butyldimethylsilyl)oxy)phenyl)propan-1-ol (18).*** Under nitrogen atmosphere, LiAlH_4_ (38 mg, 1.2 mmol) was added slowly to the THF (12 mL) solution of **17** (294 mg, 1 mmol) at 0 ºC and then the solution was stirred for two hours at room temperature. After that, a solution of NaOH (10% in water) was added carefully until a white solid precipitated. After filtration over MgSO_4_ and evaporation of the solvent the crude was obtained. The crude material was purified by chromatography silica gel (*n*-hexane/EtOAc) to give the pure product **18**, as a colorless solid (231 mg, 87%). ^1^H NMR (600 MHz, CDCl_3_) *δ* 7.04 (d, *J* = 8.3 Hz, 2H), 6.75 (d, *J* = 8.4 Hz, 2H), 3.66 (t, *J* = 6.4 Hz, 2H), 2.65 – 2.62 (m, 2H), 1.88 – 1.83 (m, 2H), 0.98 (s, 9H), 0.18 (s, 6H); ^13^C NMR (150 MHz, CDCl_3_) *δ* 153.8, 134.5, 129.4, 129.4, 120.1, 120.1, 62.5, 34.5, 31.4, 25.8, 25.8, 25.8, 18.3, − 4.3, − 4.3; HRMS (EI): *m/z* [M]^+^ calcd for C_15_H_26_O_2_Si: 266.1698; found: 266.1697.

***3-(4-((tert-Butyldimethylsilyl)oxy)phenyl)propanal (11).*** To a solution of **18** (372 mg, 1.4 mmol) in DCM (7 mL) was added DMP (356 mg, 1.68 mmol) at 0 ℃. The reaction mixture was stirred at room temperature for 30 min. Then, the mixture was concentrated under reduced pressure and purified by silica gel rapid column chromatography (hexane: EtOAc = 30:1) to obtain **11** (314 mg, 85%), a colorless liquid. ^1^H NMR (600 MHz, CDCl_3_) *δ* 9.81 (d, *J* = 1.6 Hz, 1H), 7.04 (d, *J* = 8.3 Hz, 2H), 6.76 (d, *J* = 8.2 Hz, 2H), 2.89 (t, *J* = 7.5 Hz, 2H), 2.74 (td, *J* = 7.6, 1.6 Hz, 2H), 0.97 (s, 9H), 0.18 (s, 6H); ^13^C NMR (150 MHz, CDCl_3_) *δ* 202.2, 154.3, 133.1, 129.4, 129.4, 120.3, 120.3, 45.7, 27.6, 25.9, 25.9, 25.9, 18.4, − 4.2, − 4.2; HRMS (EI): *m/z* [M]^+^ calcd for C_15_H_24_O_2_Si: 264.15401; found: 264.15387.

## 3.Synthesis of 5

***Methyl (E)-3-(4-methoxyphenyl)acrylate(20).*** To a stirred solution of **19** (356 mg, 2 mmol) and iodomethane (250 *µ*L, 4 mmol) in DMF (10 mL) was added K_2_CO_3_ (337 mg, 6 mmol). The resultant suspension was heated at 60 °C for 4 h, allowed to cool to rt and stirred overnight. The reaction mixture was filtered, the cake washed with EtOAc (30 mL) and the filtrate washed with brine (3 × 20 mL). The organic layer was dried over MgSO_4_, filtered, and concentrated under reduced pressure. The crude product was purified by flash chromatography on silica gel (hexane : EtOAc/10:1) to give **20** (353 mg, 1.84 mmol, 92%) as white solid .^1^H NMR (600 MHz, CDCl_3_) *δ* 7.32 (d, *J* = 8.7 Hz, 1H), 6.86 (d, *J* = 8.7 Hz, 1H), 4.29 (dd, *J* = 5.9, 1.5 Hz, 1H), 3.81 (s, 2H); ^13^C NMR (150 MHz, CDCl_3_) *δ* 159.4, 131.1, 129.5, 127.8, 127.8, 126.4, 114.1, 114.1, 114.1, 64.1, 55.4; HRMS (EI): *m/z* [M]^+^ calcd for C_11_H_12_O_3_: 192.0781; found: 192.0780.

***(E)-3-(4-Methoxyphenyl)prop-2-en-1-ol (21).*** Under nitrogen atmosphere, LiAlH_4_ (91.2 mg, 2.4 mmol) was added slowly to the THF (12 mL) solution of **20** (384 mg, 2 mmol) at 0 ºC and then the solution was stirred for two h at room temperature. After that, a solution of NaOH (10% in water) was added carefully until a white solid precipitated. After filtration over MgSO_4_ and evaporation of the solvent the crude amine was obtained. The crude material was purified by chromatography silica gel, (hexane/EtOAc 5:1) to give the pure product **21**, as colorless solid (288 mg, 88%). ^1^H NMR (600 MHz, CDCl_3_) *δ* 7.35 – 7.31 (m, 2H), 6.87 – 6.83 (m, 2H), 6.60 (d, *J* = 15.8 Hz, 1H), 6.15 (dt, *J* = 15.9, 6.7 Hz, 1H), 4.70 (d, *J* = 6.7, 2H), 3.81 (s, 3H); ^13^C NMR (150 MHz, CDCl_3_) *δ* 161.6, 144.8, 129.9, 129.9, 127.3, 115.5, 114.5, 114.5, 55.6, 51.8; HRMS (EI): *m/z* [M]^+^ calcd for C_10_H_12_O_2_: 164.0832; found: 164.0831.

***(E)-3-(4-Methoxyphenyl)allyl acetate (5).*** Acetic anhydride (146 *µ*L, 1.54 mmol) was added to a solution of **21** (210mg, 1.54 mmol), Et_3_N (303 *µ*L, 2.1mmol), DMAP (2 mg, 0.014 mmol) and CH_2_Cl_2_ (7 mL) at room temperature. This solution was maintained for 1 h at room temperature, and quenched with H_2_O. The mixture was extracted with Et_2_O (2 × 10 mL). The combined organic layers were washed with brine (10 mL), dried over MgSO_4_, and concentrated. The residue was purified by silica gel column chromatography (hexane/EtOAc 8:1) to give **5** (259 mg, 90%).^1^H NMR (600 MHz, CDCl_3_) *δ* 7.65 (d, *J* = 15.9 Hz, 1H), 7.51 – 7.47 (m, 1H), 6.90 (d, *J* = 8.8 Hz, 2H), 6.31 (d, *J* = 15.9 Hz, 1H), 3.83 (s, 3H), 3.79 (s, 3H); ^13^C NMR (150 MHz, CDCl_3_) *δ* 171.1, 159.7, 134.2, 129.1, 128.0, 128.0, 120.9, 114.1, 114.1, 65.5, 55.4, 21.2; HRMS (EI): *m/z* [M]^+^ calcd for C_12_H_14_O_3:_ 206.09375; found: 206.09353.

## 4. Synthesis of 9

***4-((tert-Butyldimethylsilyl)oxy)benzaldehyde*** *(****22).*** Under nitrogen atmosphere, to a solution of **22** (1.0 g, 8.19 mmol) and imidazole (612 mg, 9 mmol) in DMF (15 mL) was added chloro *tert*-butyldimethylsilane (TBDMSCl, 1.35 g, 9 mmol). The resulting solution was stirred for 24 h at room temperature. The reaction mixture was diluted with hexane/Et_2_O = 1/1 (10 mL), and organic layer was washed three times with H_2_O (10 mL) and once with brine (10 mL), dried over Na_2_SO_4_, filtered, and the filtrate was concentrated to give a colorless liquid, which crystallized on standing to yield as a yellow solid **23** (847 mg, 95%).^1^H NMR (600 MHz, CDCl_3_) *δ* 9.87 (s, 1H), 7.78 (d, *J* = 8.6 Hz, 2H), 6.93 (d, *J* = 8.6 Hz, 2H), 0.98 (s, 9H), 0.24 (s, 6H); ^13^C NMR (150 MHz, CDCl_3_) *δ* 191.1, 161.7, 132.1, 130.5, 120.6, 25.7, 25.7, 25.7, 18.4, − 4.3, − 4.3; HRMS (ESI): *m/z* [M + H]^+^ calcd for C_13_H_20_O_2_Si_:_ 237.1305; found: 237.1309.

***1-(4-((tert-Butyldimethylsilyl)oxy)phenyl)but-3-en-1-ol (24).*** To a solution of **23** (476 mg, 2.02 mmol) in THF (20 mL), 1.0 M allylmagnesium bromide solution (3 mL, 3 mmol) was added at 0 °C until the starting aldehyde disappeared based on TLC analysis. The mixture was poured onto saturated aqueous NH_4_Cl (5 mL) and extracted with AcOEt (20 mL × 3). The organic extracts were washed with brine, dried over MgSO_4,_ and then concentrated in vacuo. Silica gel column chromatography of the residue (hexane: AcOEt/10:1) gave **24** (420 mg, 75%) as a yellow solid. ^1^H NMR (600 MHz, CDCl_3_) *δ* 7.21 (d, *J* = 8.2 Hz, 2H), 6.81 (d, *J* = 8.4 Hz, 2H), 5.79 (ddt, *J* = 17.2, 10.3, 7.2 Hz, 1H), 5.17 – 5.11 (m, 2H), 4.67 (t, *J* = 6.6 Hz, 1H), 2.49 (t, *J* = 6.9 Hz, 2H), 0.98 (s, 9H), 0.19 (s, 6H); ^13^C NMR (151 MHz, CDCl_3_) *δ* 155.2, 136.8, 134.8, 127.1, 127.1, 120.1, 120.1, 118.3, 73.2, 43.9, 25.8, 25.8, 25.8, 18.3, − 4.3, − 4.3; HRMS (ESI): *m/z* [M + Na]^+^ calcd for C_16_H_24_O_2_Si_:_ 301.1594; found: 301.1592.

***(4-(But-3-en-1-yl)phenoxy)(tert-butyl)dimethylsilane (25)***. To a solution of **24 (**278 mg, 1.0 mmol) and Et_3_SiH (303 *µ*L, 2 mmol) in CH_2_Cl_2_ (5 mL) was added BF_3_·Et_2_O (123 *µ*L, 1 mmol) at 0 °C. After the reaction mixture was stirred at 0 °C for 2 h, saturated aqueous NaHCO_3_ solution was added. The organic solution was separated, washed with brine, and dried (anhydr. Na_2_SO_4_). The solvent was concentrated, and then the residue was subjected to silica gel column chromatography (hexane) to give **25** (207 mg, 79%) as a colorless liquid. ^1^H NMR (600 MHz, CDCl_3_) *δ* 7.03 (d, *J* = 8.2 Hz, 2H), 6.75 (d, *J* = 8.3 Hz, 2H), 5.85 (dt, *J* = 16.9 Hz, 1H), 5.09 – 4.90 (m, 2H), 2.63 (dd, *J* = 9.0, 6.7 Hz, 2H), 2.33 (m, *J* = 7.2, 6.5, 1.4 Hz, 2H), 0.98 (s, 9H), 0.18 (s, 6H); ^13^C NMR (150 MHz, CDCl_3_) *δ* 153.8, 138.4, 134.7, 129.4, 129.4, 119.9, 119.9, 114.9, 35.9, 34.7, 25.9, 25.85, 25.85, 18.34, − 4.28, − 4.28; HRMS (ESI); *m/z* [M + H]^+^ calcd. for C_16_H_24_OSi_:_ 263.1826; found: 263.1825.

***4-(But-3-en-1-yl)phenol (9)***. 1.0 M TBAF (4 mL, 4.0 mmol) was added to a THF (20 mL) solution of **25** (350 mg, 1.33 mmol) at room temperature, and stirring was continued for 1 h. After complete conversion (Monitored by TLC), it was quenched by addition of saturated aq. NH_4_Cl solution. The layers were separated, and the aqueous layer was extracted with ethyl acetate (3 × 20 mL). The combined organic extracts were washed with brine (50 mL), dried (Anhydrous Na_2_SO_4_), and concentrated in vacuo. The crude product was purified by flash column chromatography on silica gel to give **9** (195 mg, 99%) as a colorless liquid. ^1^H NMR (600 MHz, CDCl_3_) *δ* 7.05 (d, *J* = 8.5 Hz, 2H), 6.75 (d, *J* = 8.4 Hz, 2H), 5.85 (dt, *J* = 16.9, 10.2, 6.6 Hz, 1H), 5.08 – 4.92 (m, 2H), 2.64 (dd, *J* = 8.9, 6.8, 2H), 2.36 – 2.29 (m, 2H); ^13^C NMR (150 MHz, CDCl_3_) *δ* 153.8, 138.3, 134.2, 129.6, 129.6, 115.2, 115.2, 115.0, 35.9, 34.6; HRMS (ESI): *m/z* [M − H]^−^ calcd. for C_16_H_24_OSi_:_ 147.0815; found: 147.0815.

## 5. Synthesis of 10

***3-Bromo-4-hydroxybenzaldehyde (26).*** PTSA (168 mg, 0.98 mmol) was added to a solution of **22** (1.20 g, 9.8 mmol) in MeCN (30 mL) at room temperature, 5 min later NBS (2.09 g, 11.76 mmol) was added in one portion. The reaction mixture was stirred at room temperature for 20 h before being quenched by a solution of NaHCO_3_ (0.94 g, 11.2 mmol) in water (50 mL). Volatiles were removed in vacuo and the aqueous residue was extracted with EtOAc (50 mL × 3). The combined organic layers were washed with aqueous Na_2_S_2_O_3_ (5%, 20 mL × 2), water (20 mL × 2) and brine (20 mL), dried over anhydrous Na_2_SO_4_ and concentrated in vacuo. The residue was purified by column chromatography (DCM) to give **26** (1.71 g, 87%) as a colorless oil. ^1^H NMR (600 MHz, CDCl_3_) *δ* 9.83 (s, 1H), 8.04 (d, *J* = 2.0 Hz, 1H), 7.77 (dd, *J* = 8.2, 2.0 Hz, 1H), 7.15 (d, *J* = 8.3 Hz, 1H).; ^13^C NMR (150 MHz, CDCl_3_) *δ* 189.6, 157.6, 134.1, 131.6, 131.2, 116.7, 111.3; HRMS (ESI): *m/z* [M − H]^−^ calcd for C_7_H_5_BrO_2_: 198.9400; found: 198.9401.

***3-Bromo-4-methoxybenzaldehyde (10).*** Compound **10** was prepared from **27** (1.7 g, 8.47 mmol) in 91.6% yield exactly following the procedure described for the preparation of **20**; ^1^H NMR (600 MHz, CDCl_3_) *δ* 9.84 (s, 1H), 8.08 (d, *J* = 2.0 Hz, 1H), 7.82 (dd, *J* = 8.7, 2.0 Hz, 1H), 7.01 (d, *J* = 8.6 Hz, 1H), 3.99 (s, 3H); ^13^C NMR (151 MHz, CDCl_3_) *δ* 189.7, 160.8, 134.7, 131.3, 130.9, 112.8, 111.7, 56.8; HRMS (ESI): *m/z* [M + H]^+^ calcd. for C_8_H_7_BrO_2:_ 214.9702; found: 214.9702.

## 6. NMR spectra copies for all synthetic compounds

**
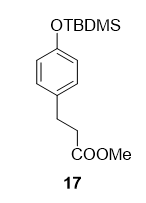
**
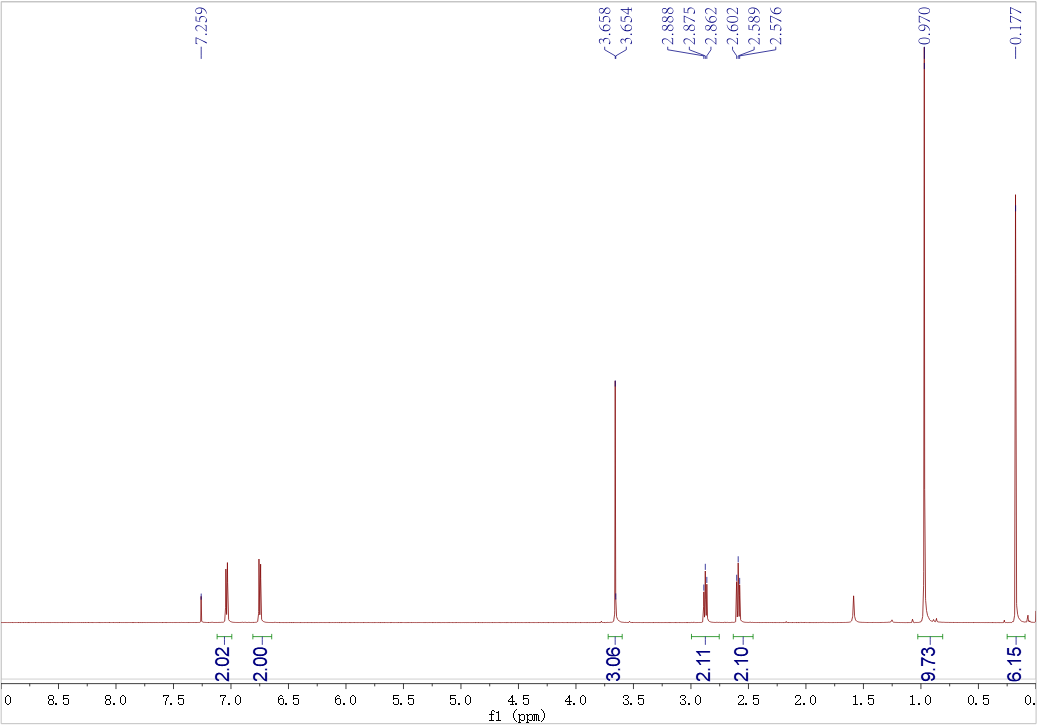


### Fig. S1. ^1^H NMR spectrum of compound 17 (CDCl_3_, 600MHz)

**
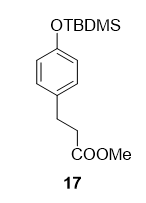
**
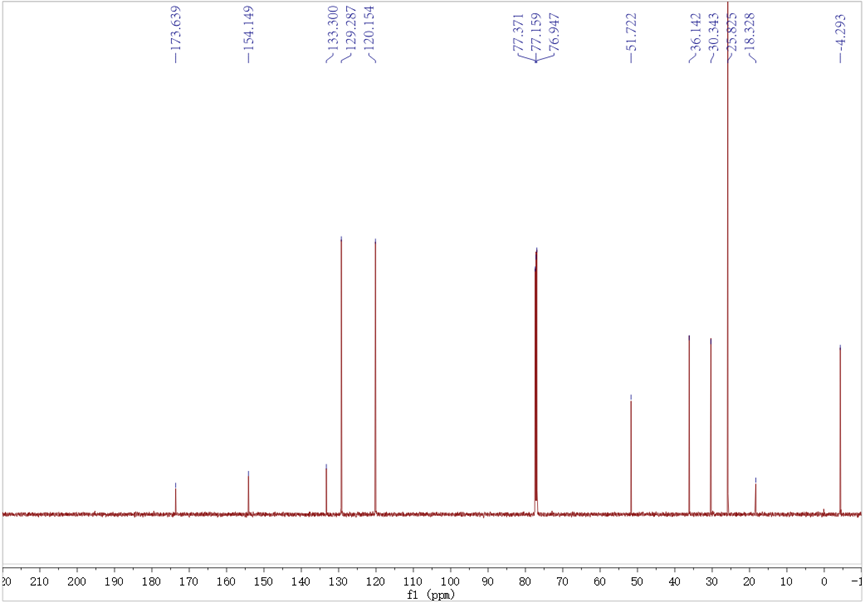


### Fig. S2. ^13^C NMR spectrum of compound 17 (CDCl_3_, 150 MHz)


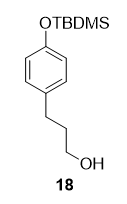

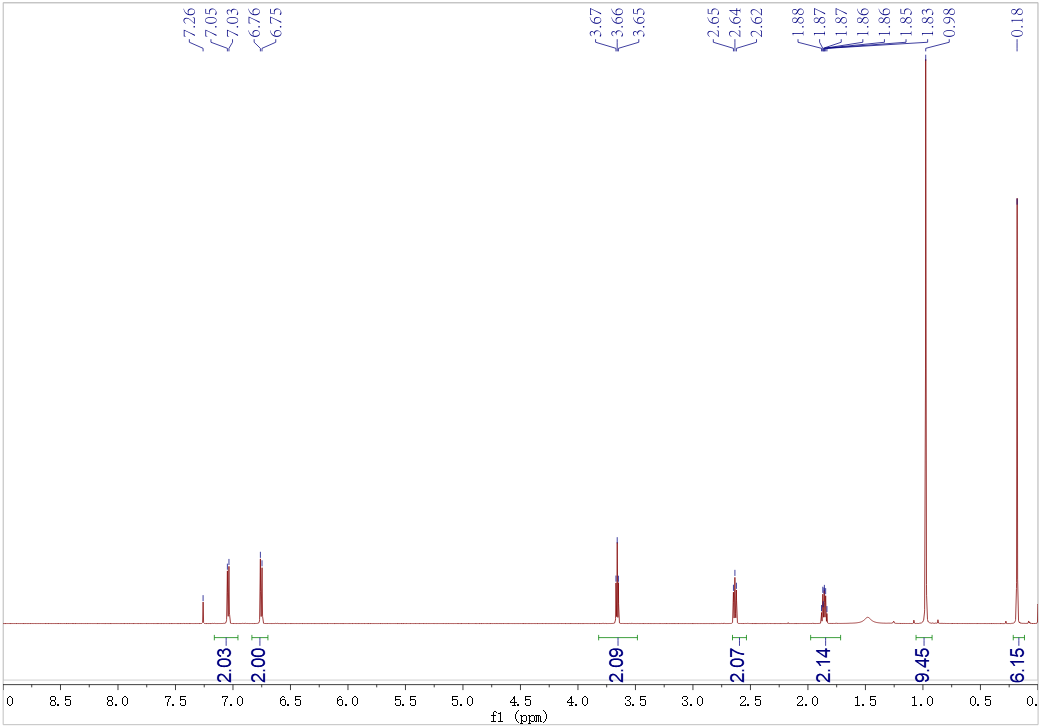


### Fig. S3. ^1^H NMR spectrum of compound 18 (CDCl_3_, 600 MHz)


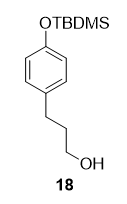
**
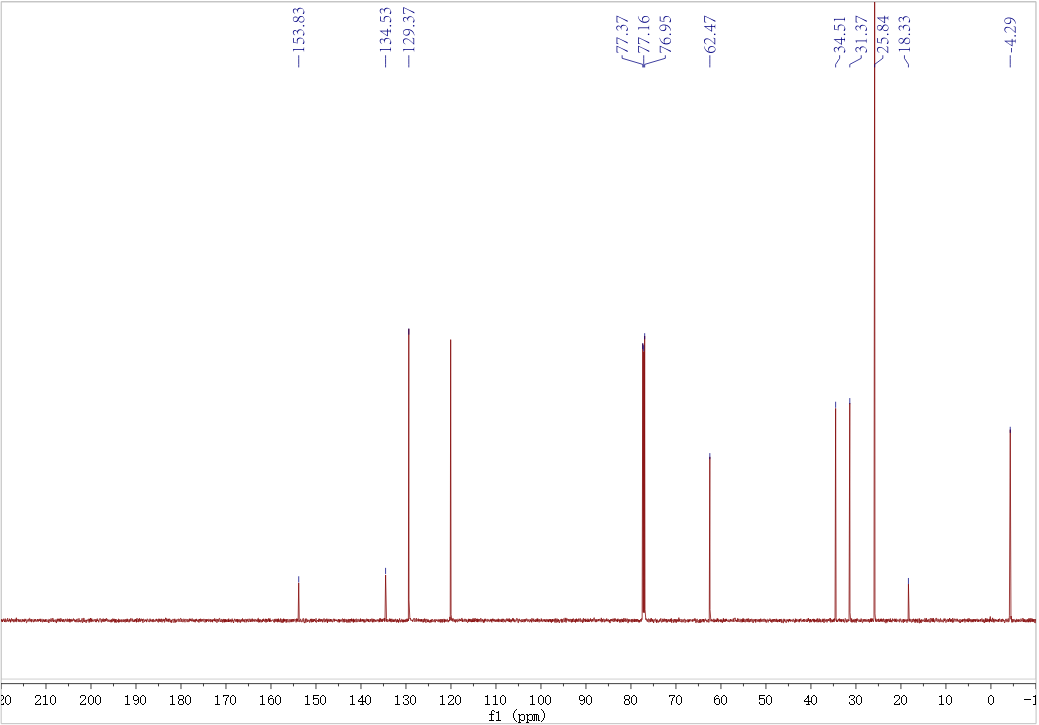
**

### Fig. S4. ^13^C NMR spectrum of compound 18 (CDCl_3_, 150 MHz)

**
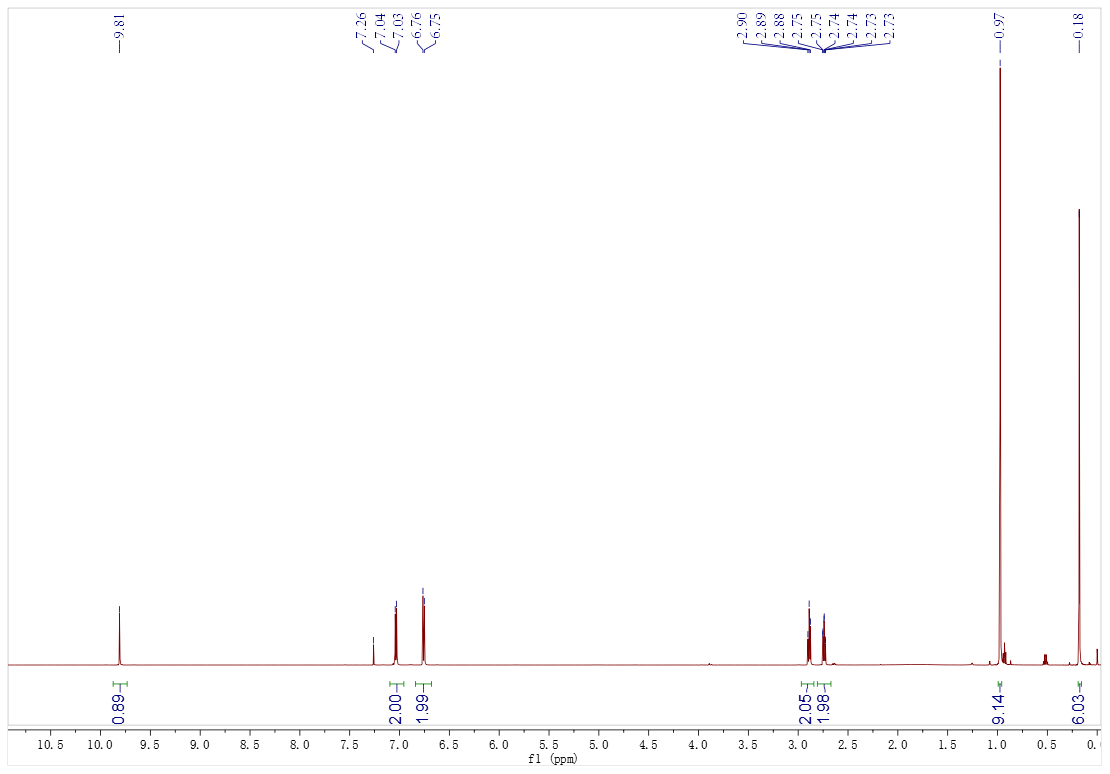
**

### Fig. S5. ^1^H NMR spectrum of compound 11 (CDCl_3_, 600 MHz)

**
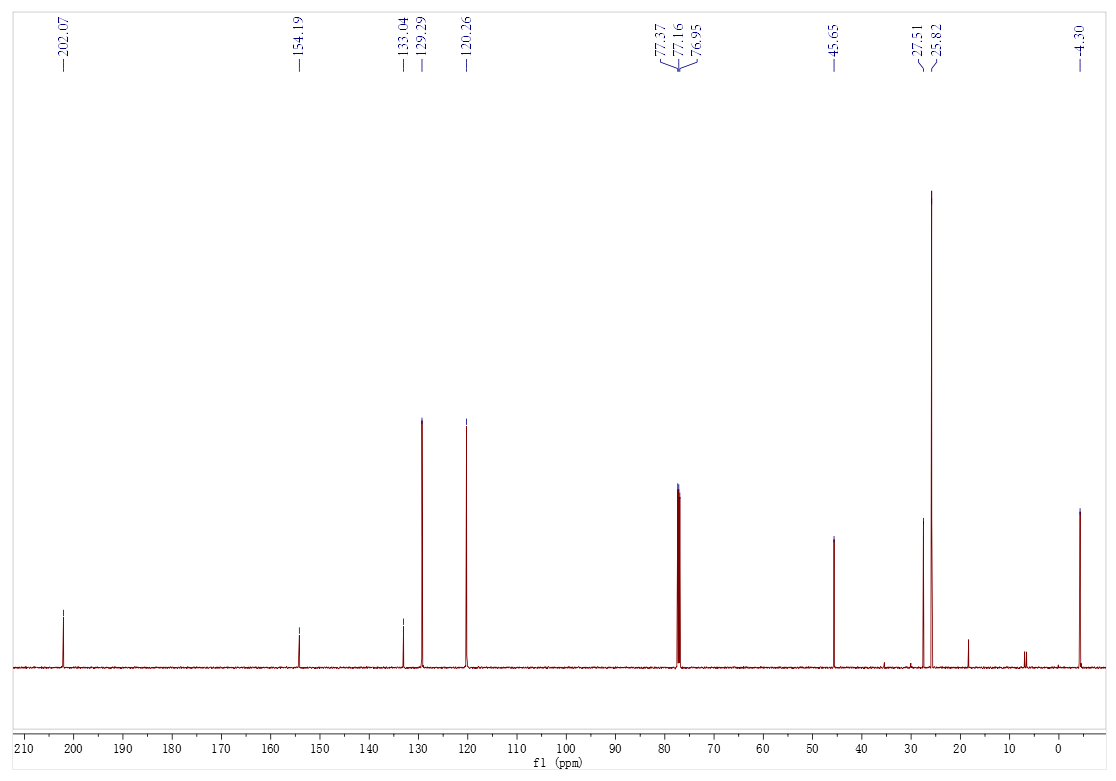
**

### Fig. S6. ^13^C NMR spectrum of compound 11 (CDCl_3_, 150 MHz)


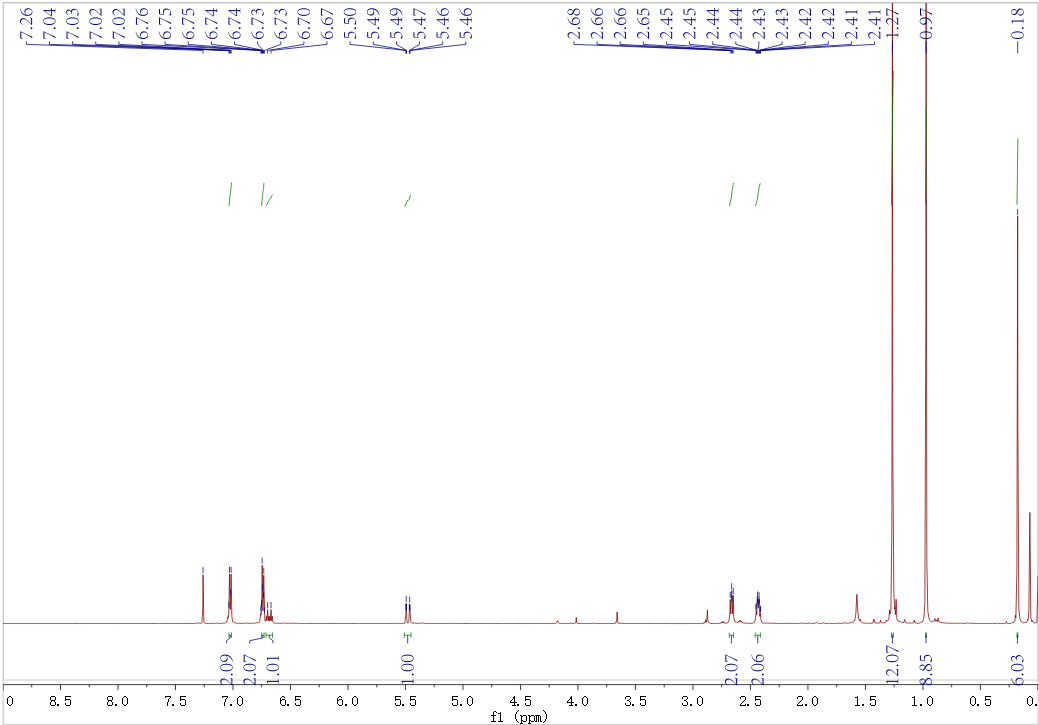


### Fig. S7. ^1^H NMR spectrum of compound 12 (CDCl_3_, 600 MHz)

**
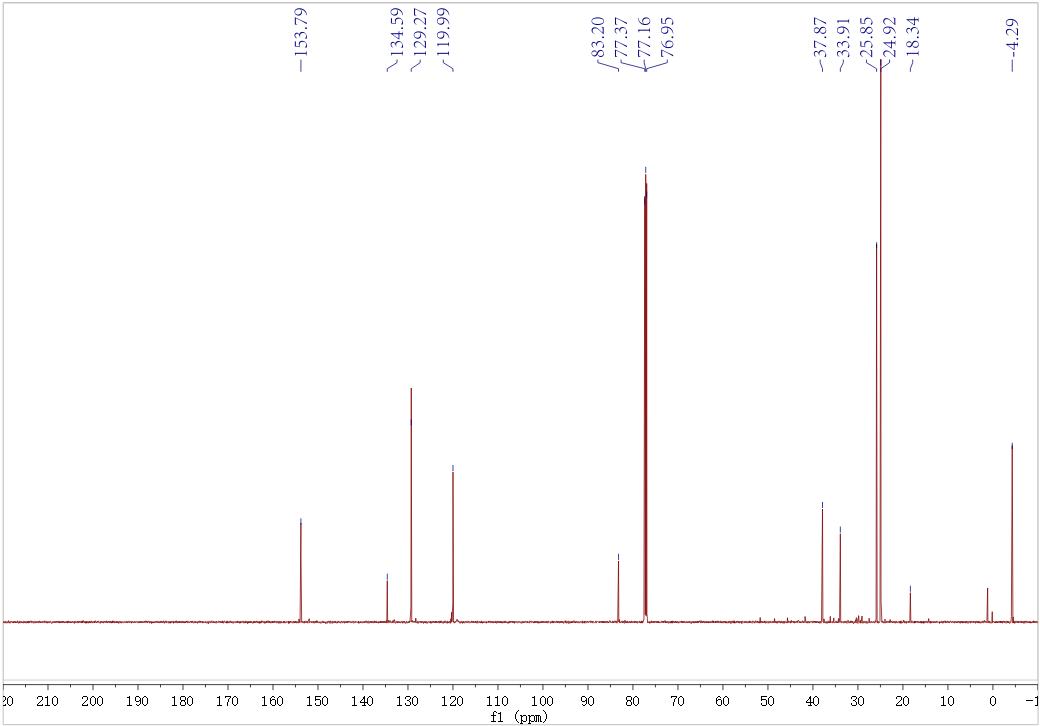
**

### Fig. S8. ^13^C NMR spectrum of compound 12 (CDCl_3_, 150 MHz)

**
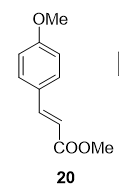

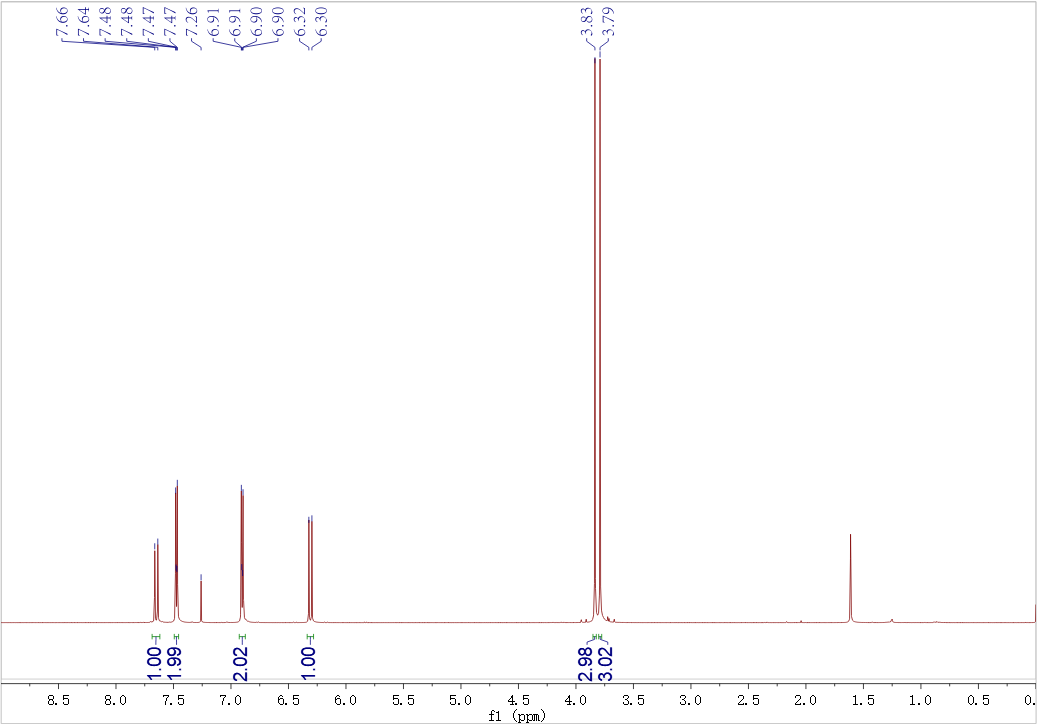
**

### Fig. S9. ^1^H NMR spectrum of compound 20 (CDCl_3_, 600MHz)

**
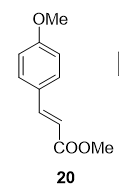

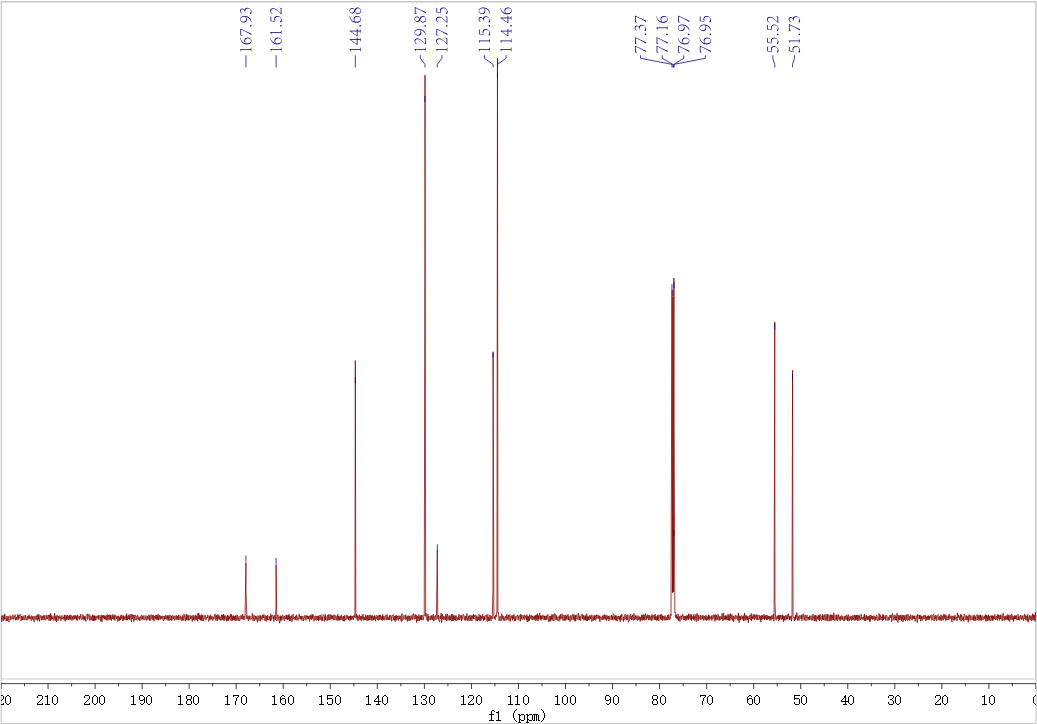
**

### Fig. S10. ^13^C NMR spectrum of compound 20 (CDCl_3_, 150 MHz)

**
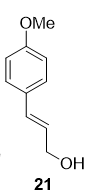

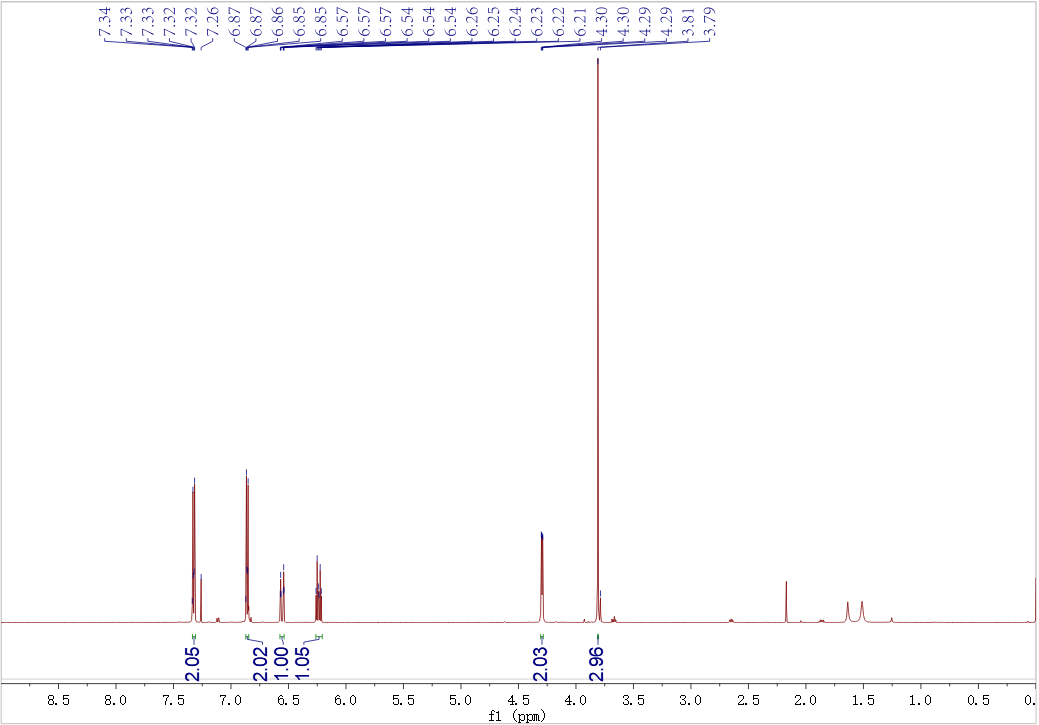
**

### Fig. S11. ^1^H NMR spectrum of compound 21 (CDCl_3_, 600 MHz)

**
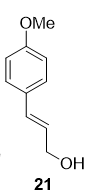

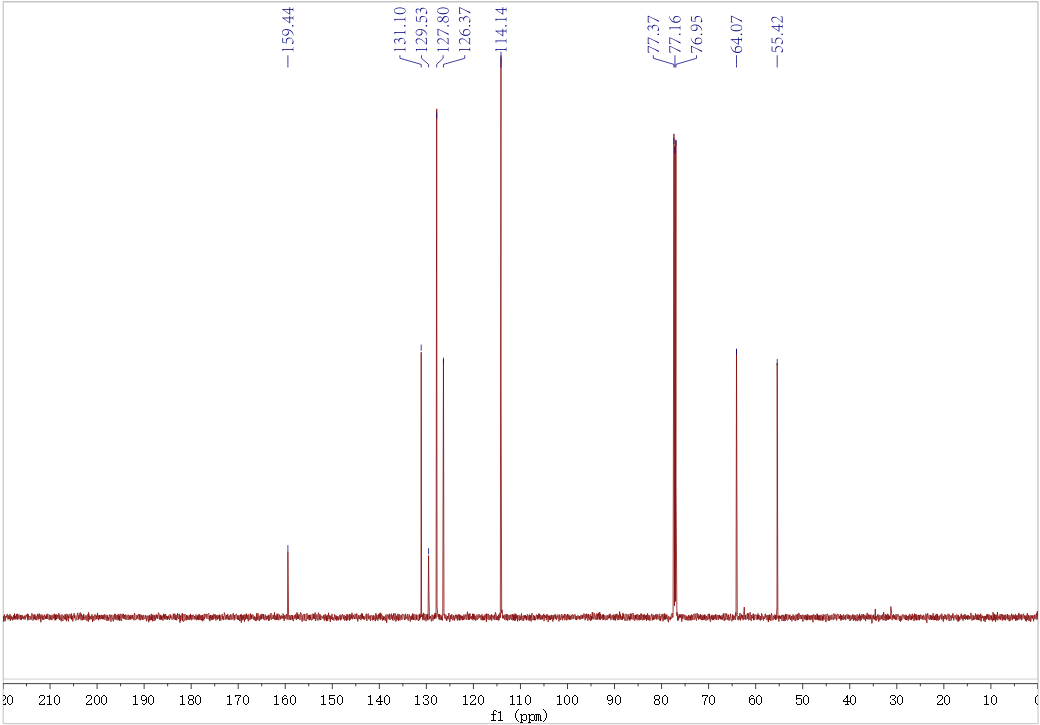
**

### Fig. S12. ^13^C NMR spectrum of compound 21 (CDCl_3_, 150 MHz)

**
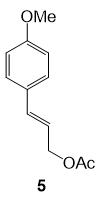

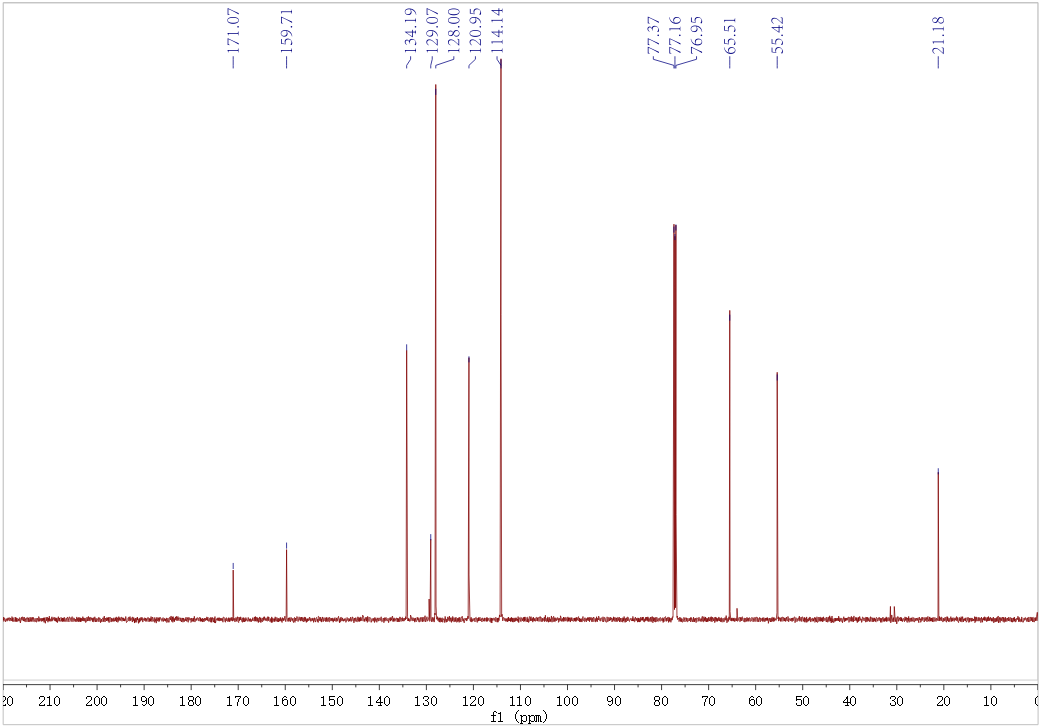
**

### Fig. S13. ^1^H NMR spectrum of compound 5 (CDCl_3_, 600 MHz)

**
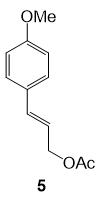

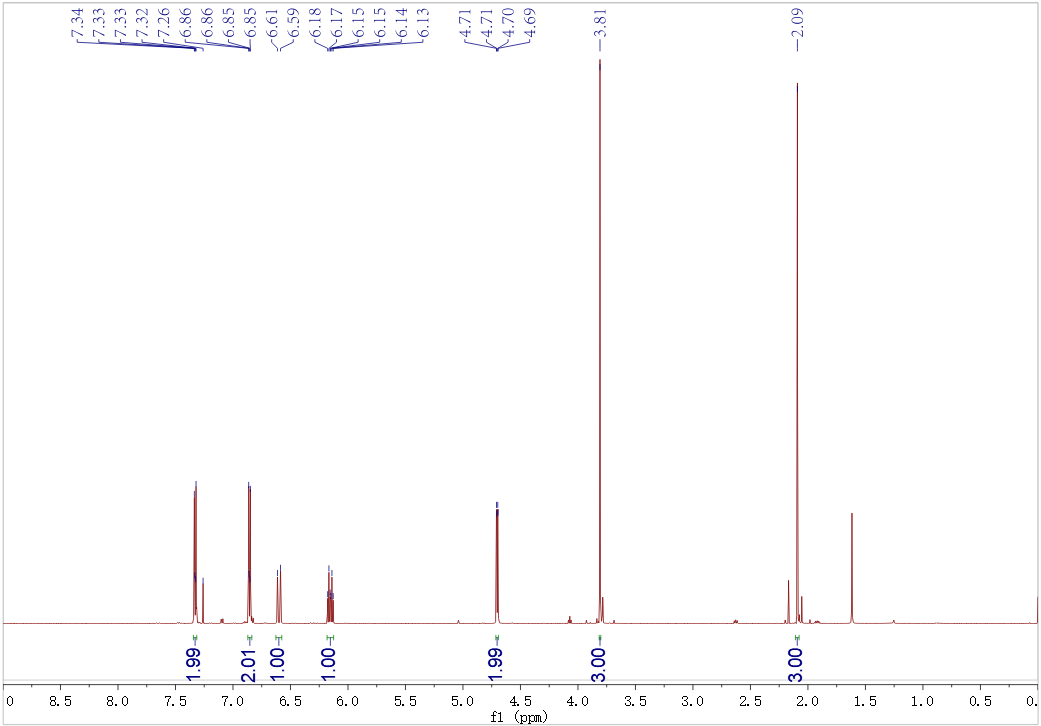
**

### Fig. S14. ^13^C NMR spectrum of compound 5 (CDCl_3_, 150 MHz)


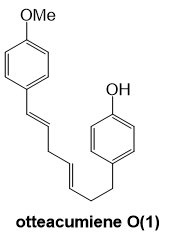
**
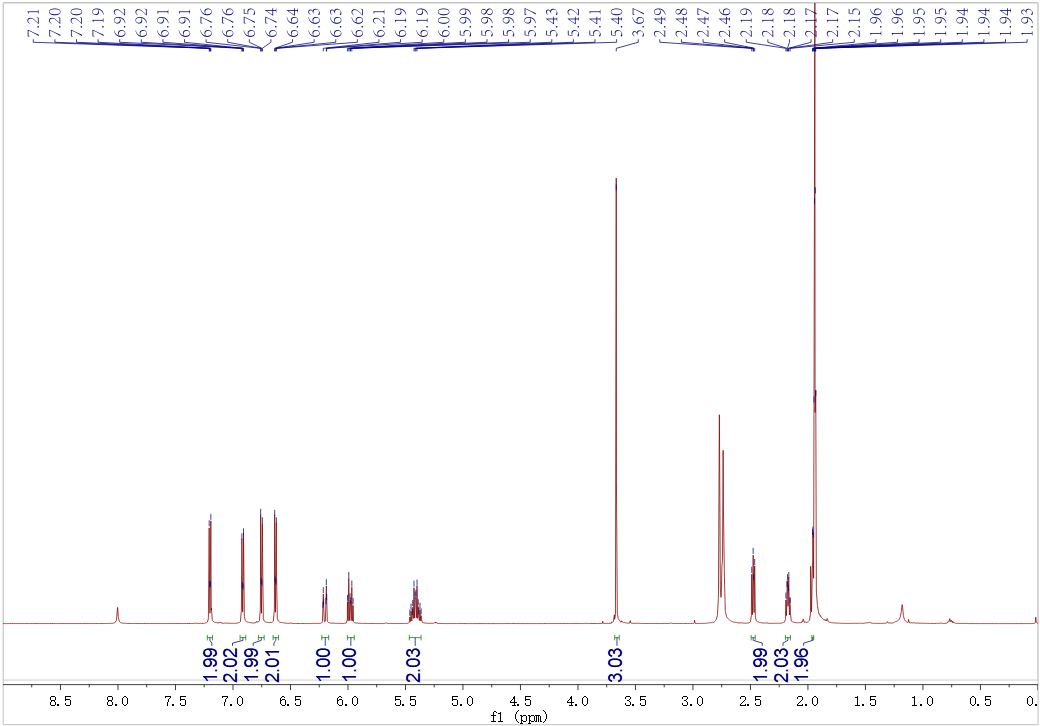
**

### **Fig. S15.** ^1^H NMR spectrum of compound **1** (Acetone-*d*_6_, 600 MHz)


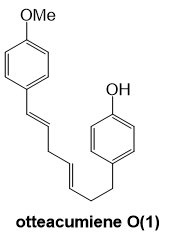
**
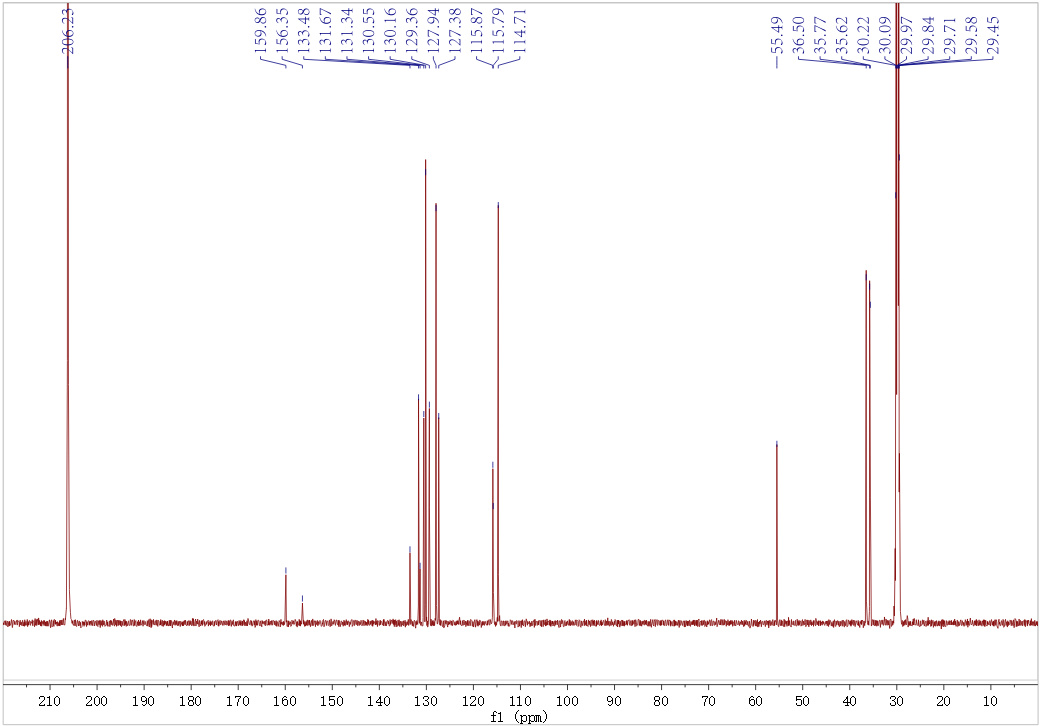
**

### Fig. S16. ^13^C NMR spectrum of compound 1 (Acetone-*d*_6_, 150 MHz)

**
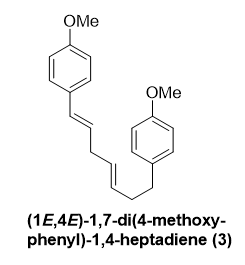
**
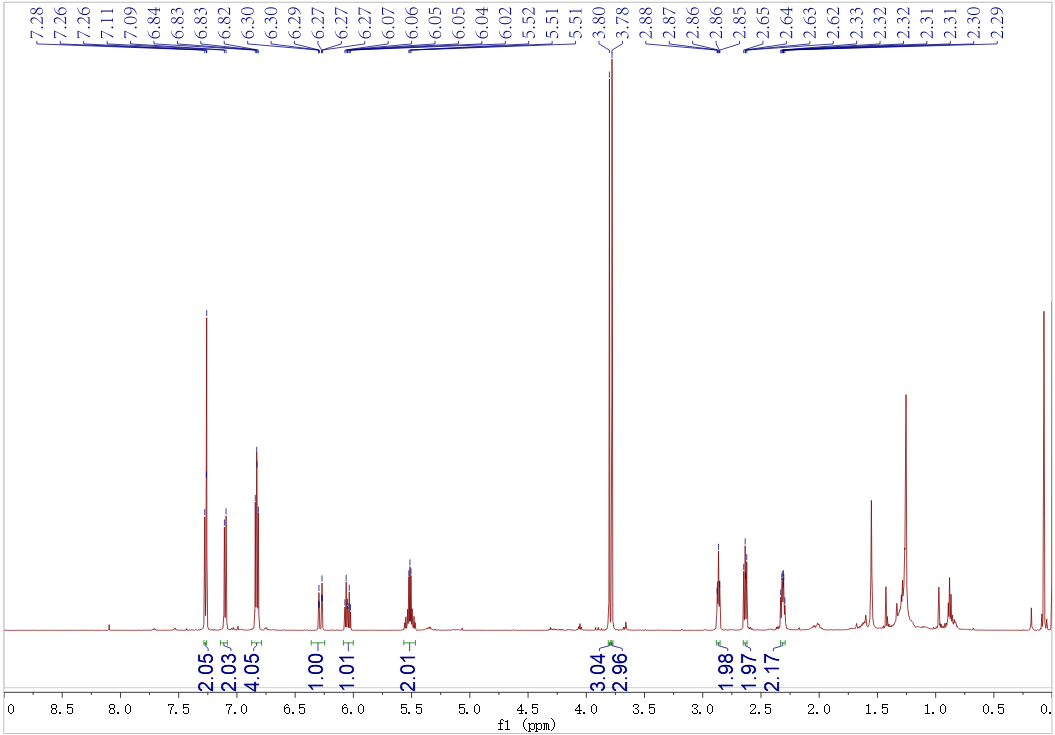


### Fig. S17. ^1^H NMR spectrum of compound 3 (CDCl_3_, 600 MHz)

**
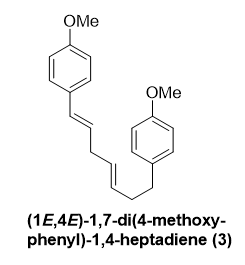

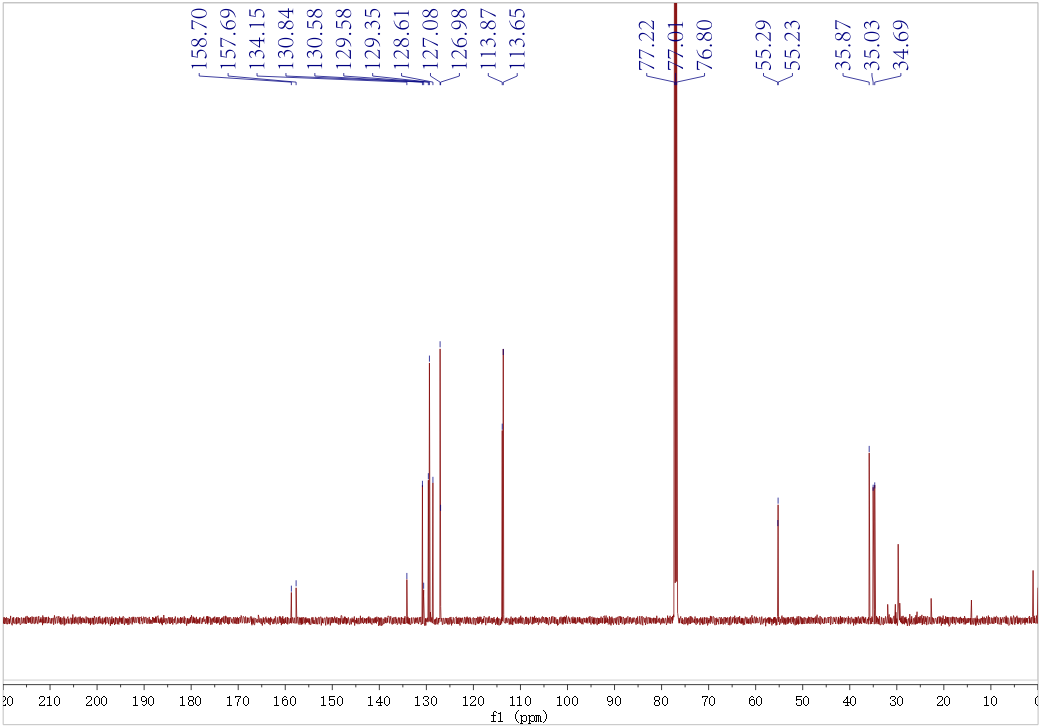
**

### Fig. S18. ^13^C NMR spectrum of compound 3 (CDCl_3_, 150 MHz)

**
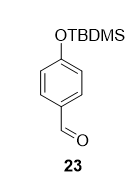

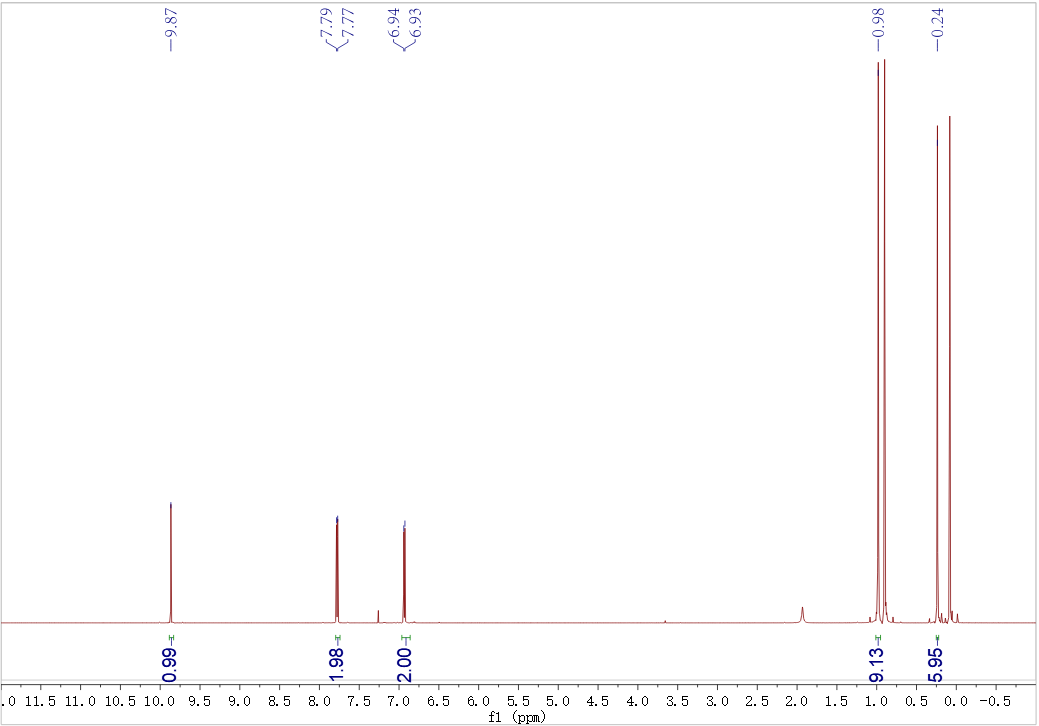
**

### **Fig. S19.** ^1^H NMR spectrum of compound **23** (CDCl_3_, 600 MHz)

**
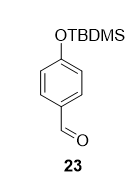

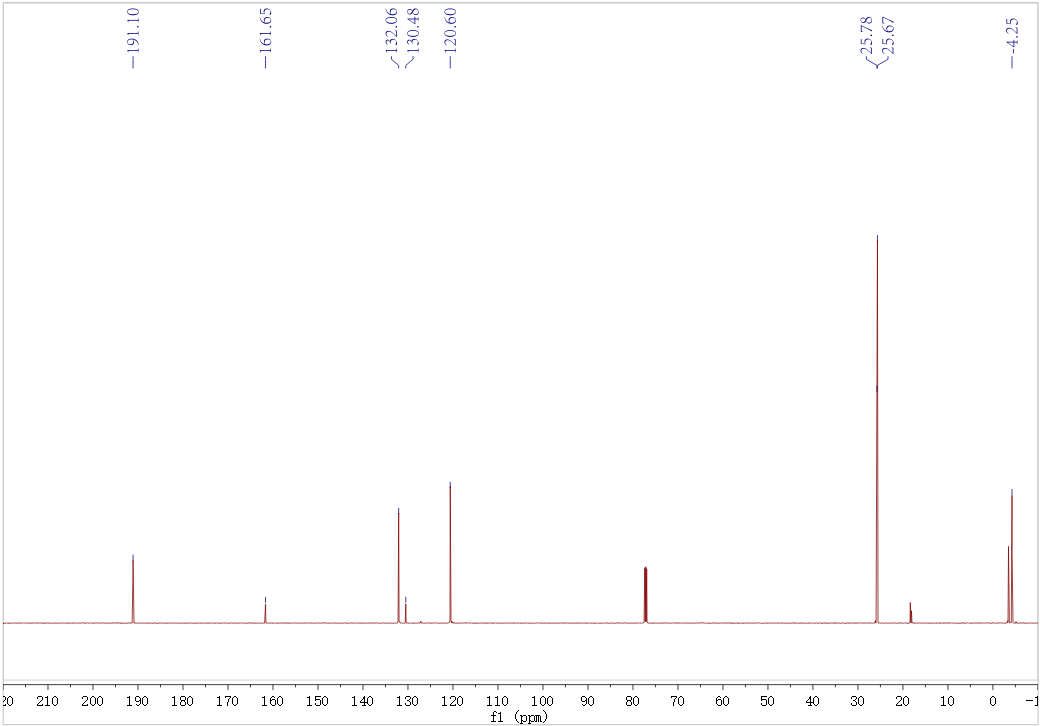
**

### Fig. S20. ^13^C NMR spectrum of compound 23 (CDCl_3_, 150 MHz)


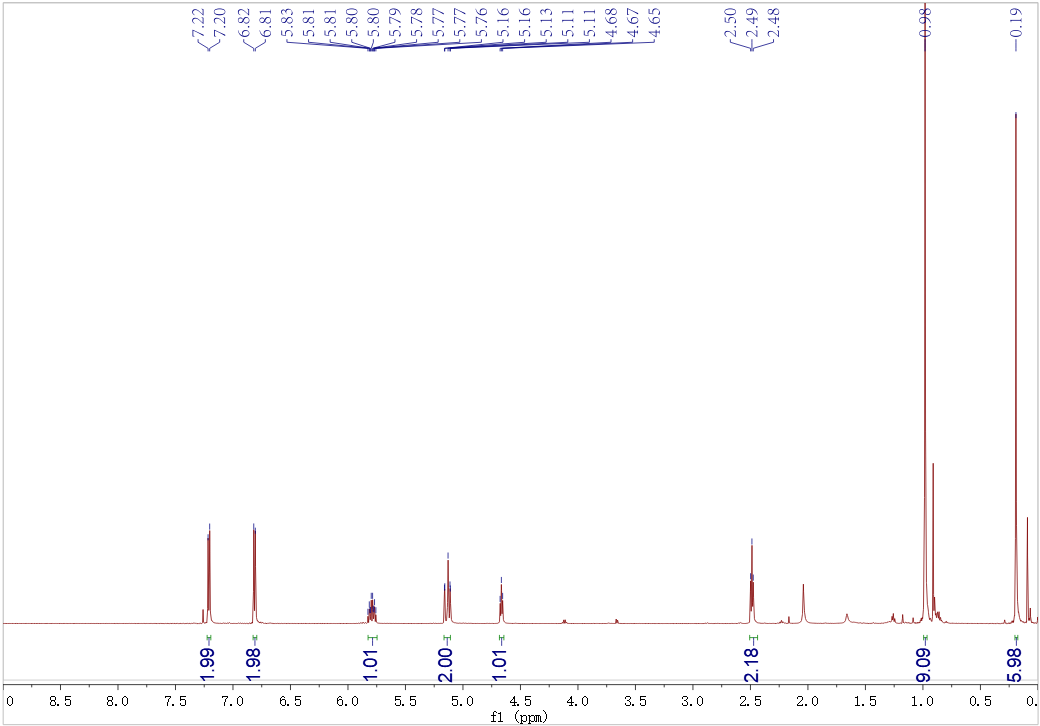


### Fig. S21. ^1^H NMR spectrum of compound 24 (CDCl**_3_**, 600 MHz)


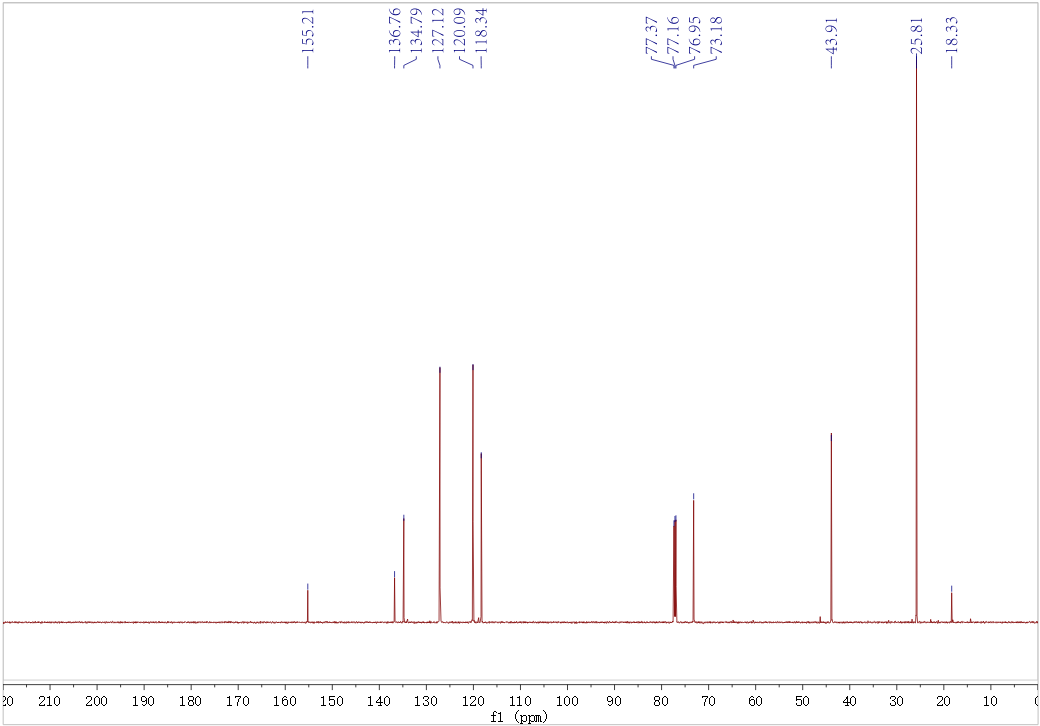


### Fig. S22. ^13^C NMR spectrum of compound 24 (CDCl**_3_**, 150 MHz)

**
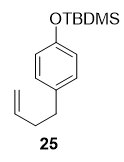
**
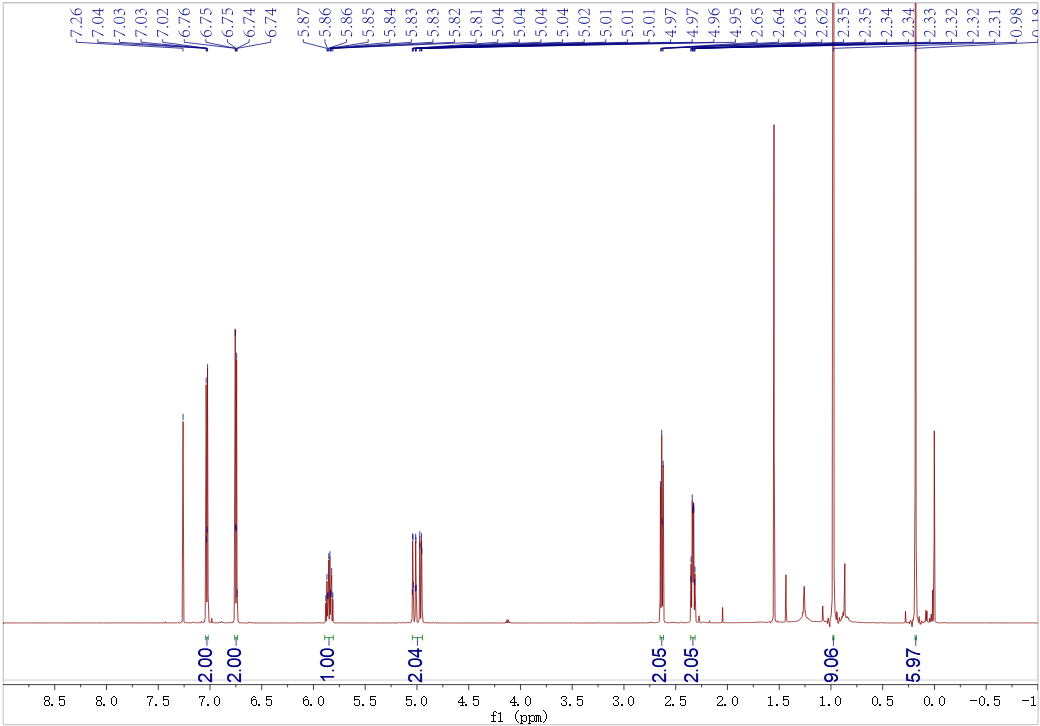


### Fig. S23. ^1^H NMR spectrum of compound 25 (CDCl**_3_**, 600 MHz)

**
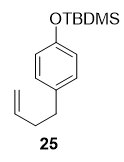
**
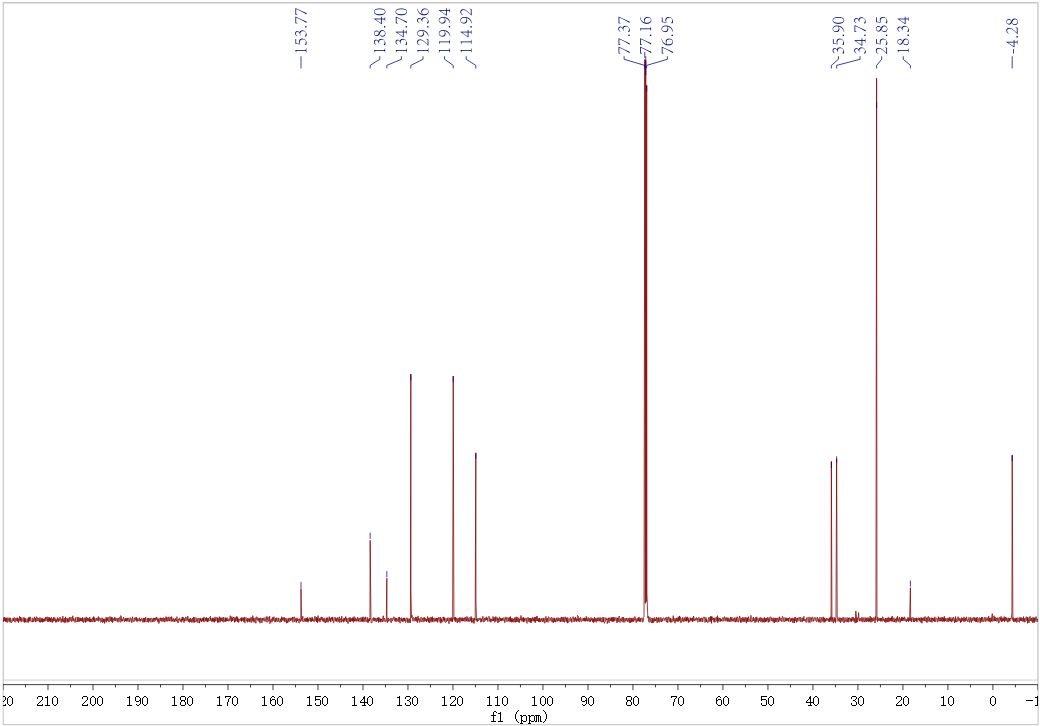


### Fig. S24. ^13^C NMR spectrum of compound 25 (CDCl**_3_**, 150 MHz)


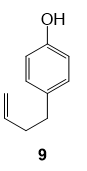

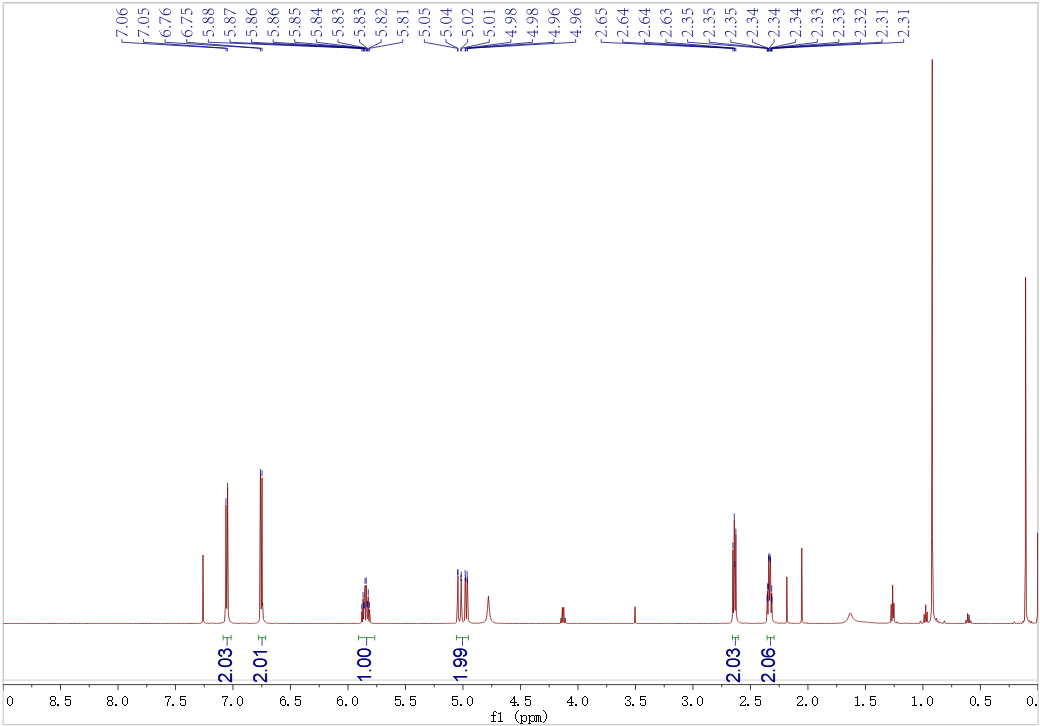


### Fig. S25. ^1^H NMR spectrum of compound 9 (CDCl**_3_**, 600 MHz)


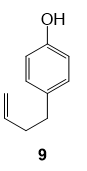

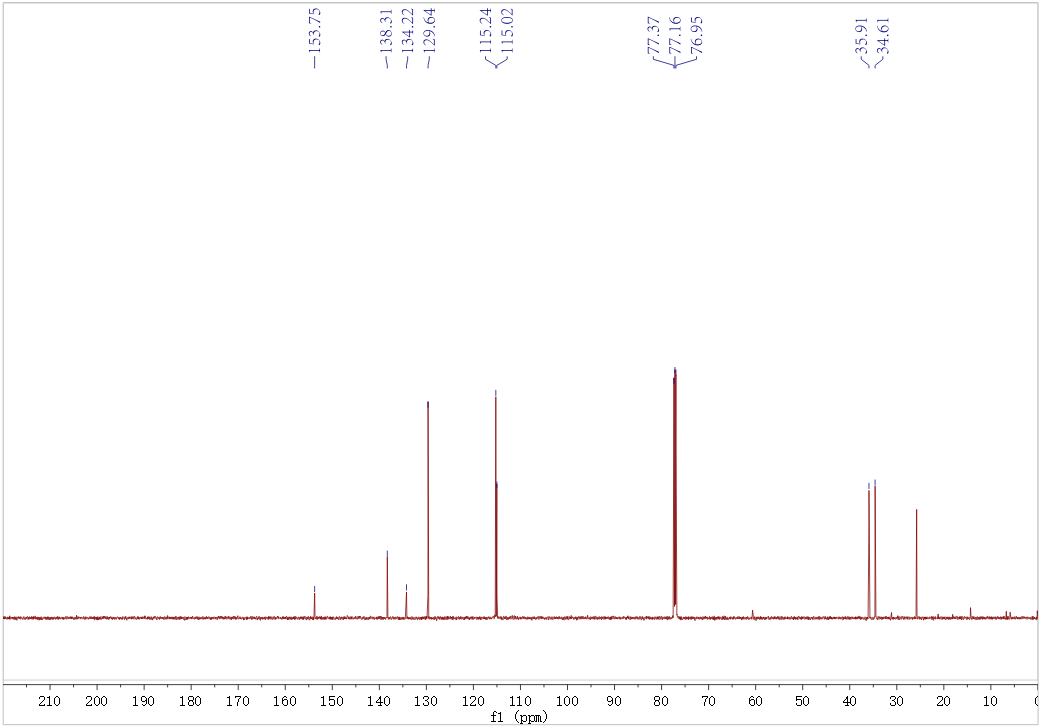


### Fig. S26. ^13^C NMR spectrum of compound 9 (CDCl**_3_**, 150 MHz)

**
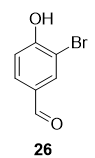
**
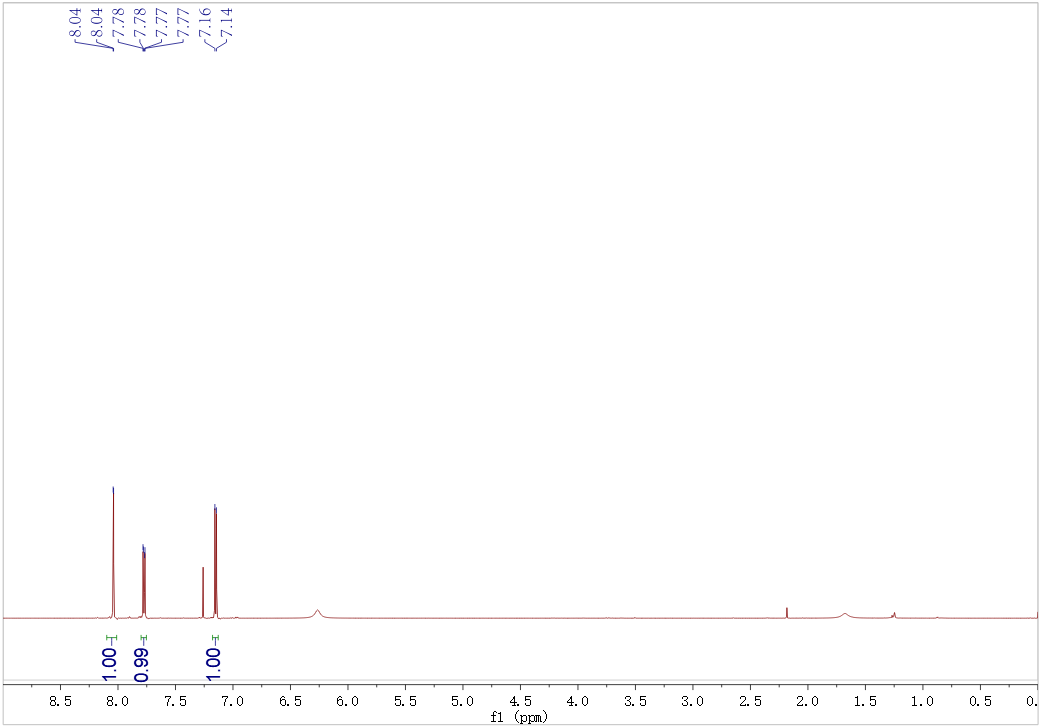


### Fig. S27. ^1^H NMR spectrum of compound 26 (CDCl**_3_**, 600 MHz)

**
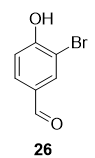
**
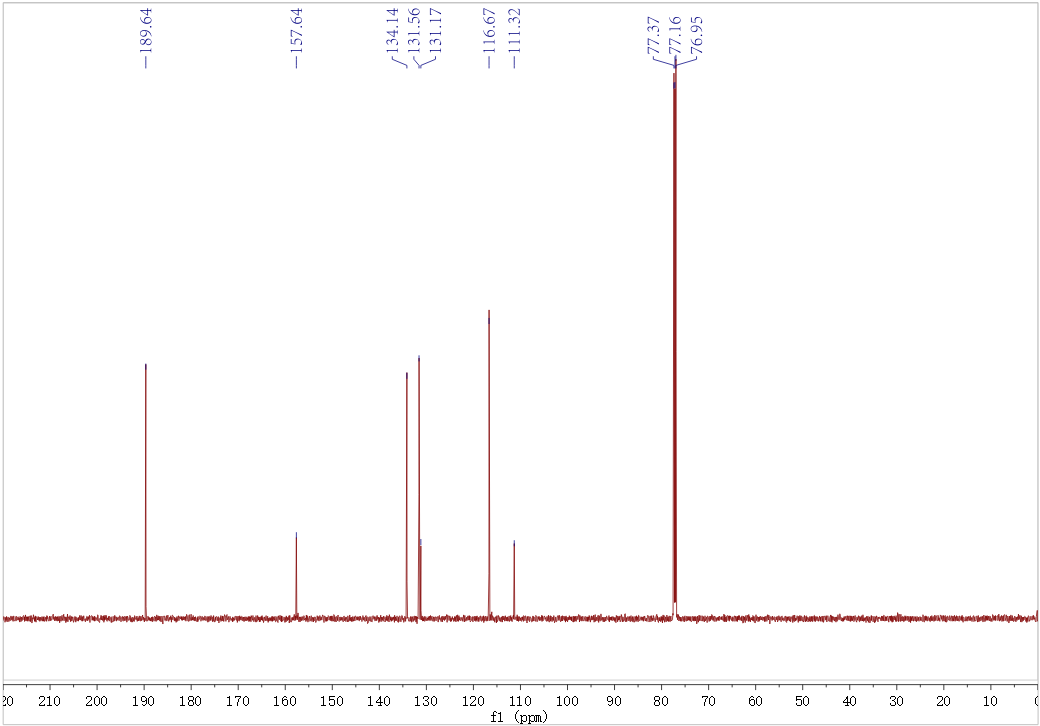


### Fig. S28. ^13^C NMR spectrum of compound 26 (CDCl**_3_**, 150 MHz)


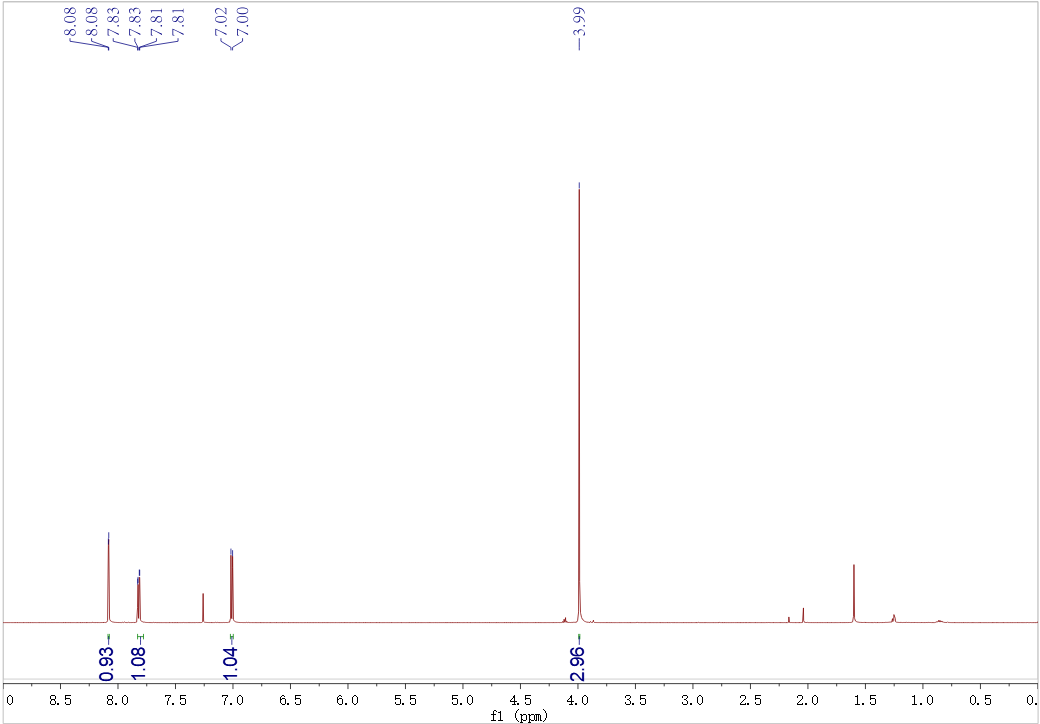


### Fig. S29. ^1^H NMR spectrum of compound 10 (CDCl**_3_**, 600 MHz)


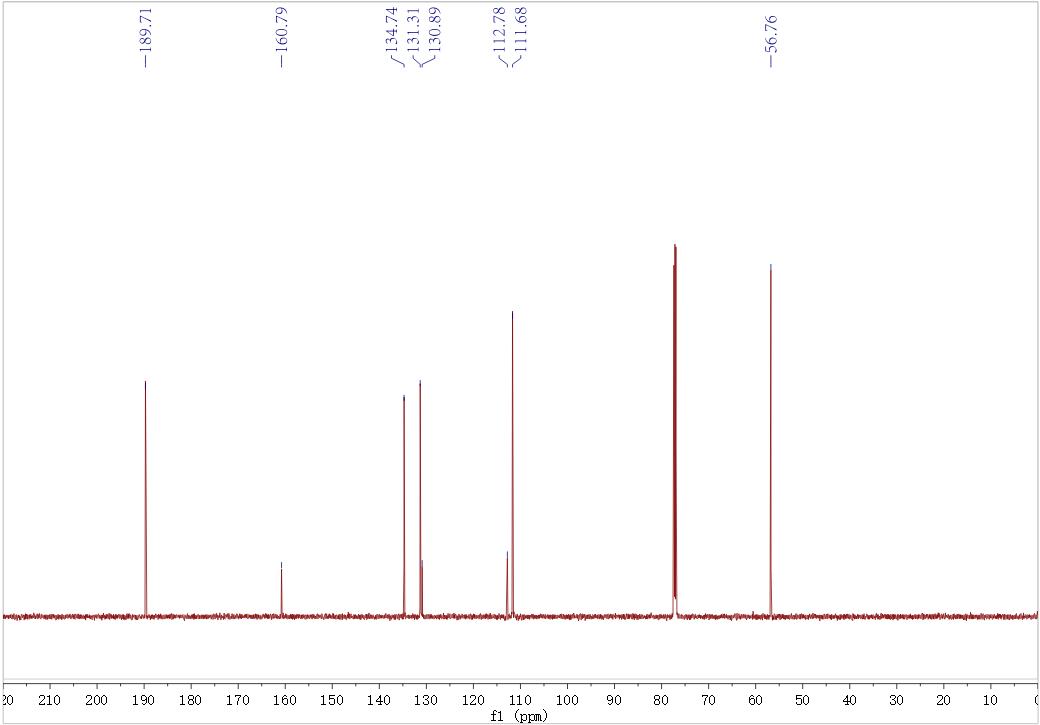


### Fig. S30. ^13^C NMR spectrum of compound 10 (CDCl**_3_**, 150 MHz)

**
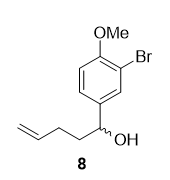
**
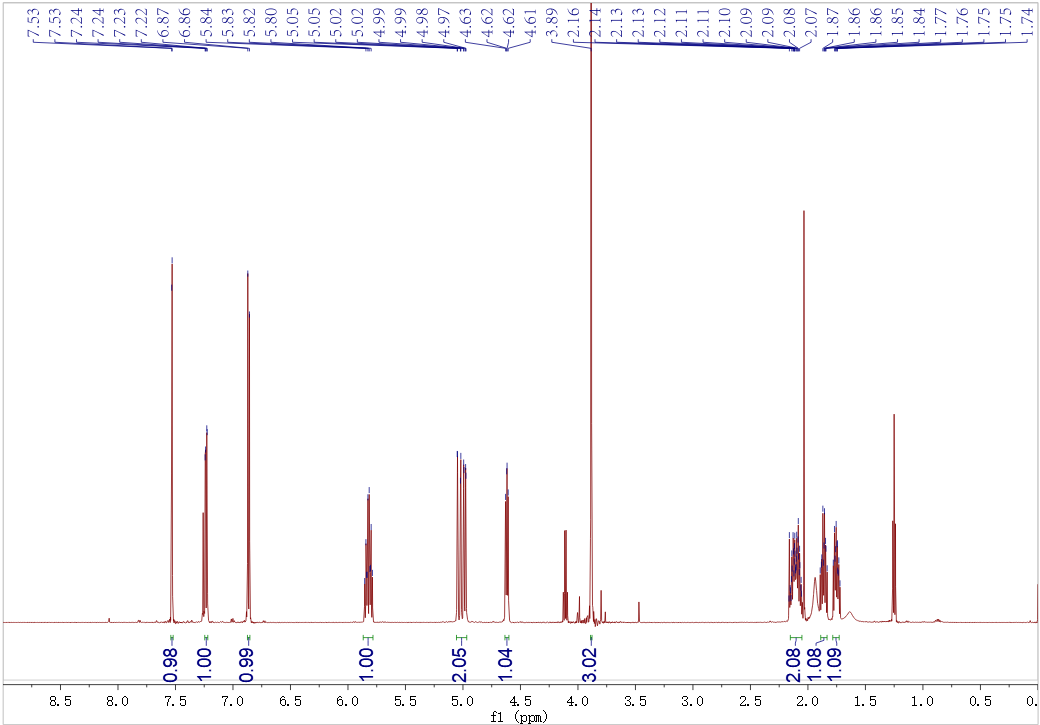


### **Fig. S31.** ^1^H NMR spectrum of compound **8** (CDCl_3_, 600 MHz)

**
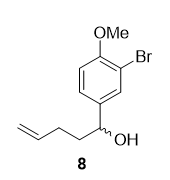
**
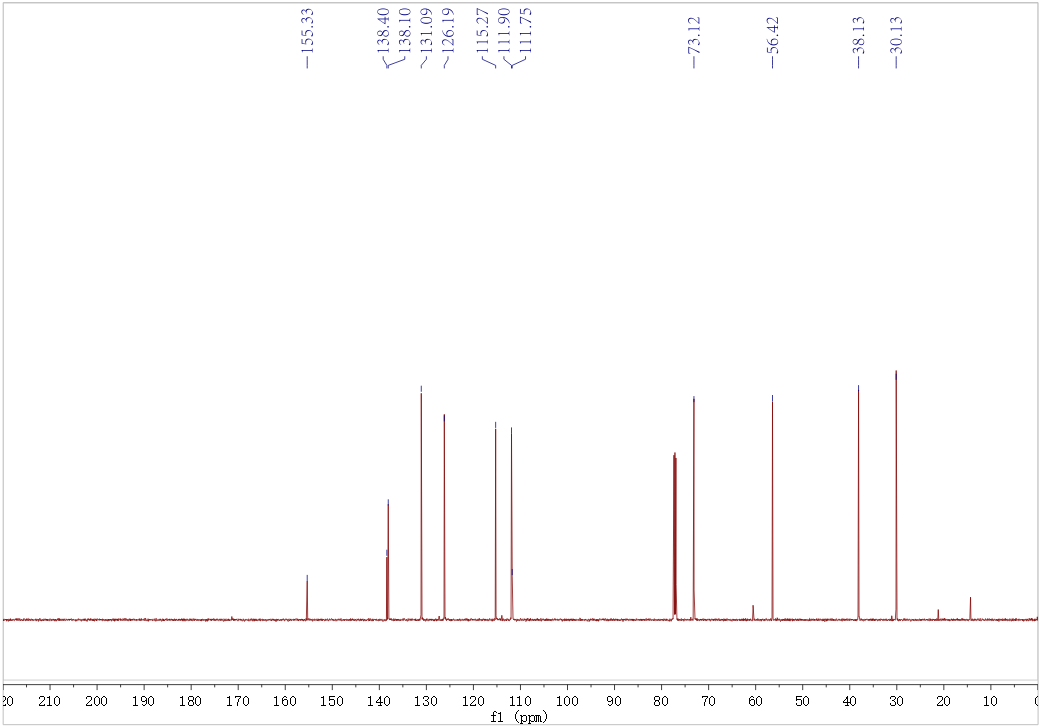


### Fig. S32. ^13^C NMR spectrum of compound 8 (CDCl**_3_**, 150 MHz)


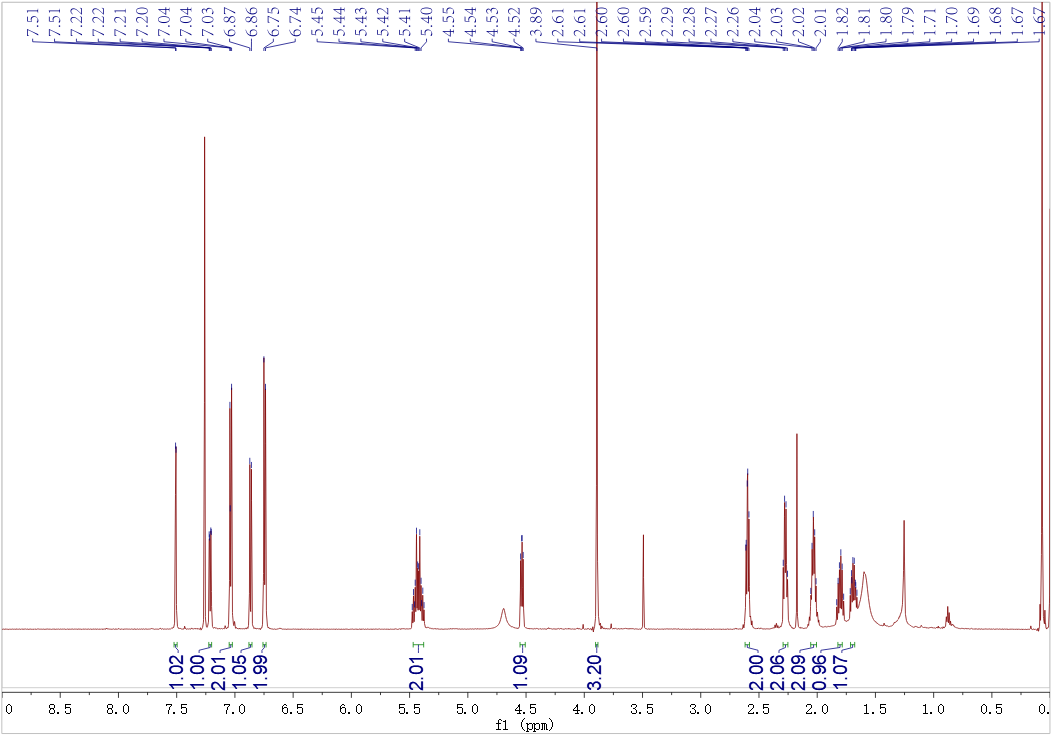


### **Fig. S33.** ^1^H NMR spectrum of compound **7-*E*** (CDCl_3_, 600 MHz)


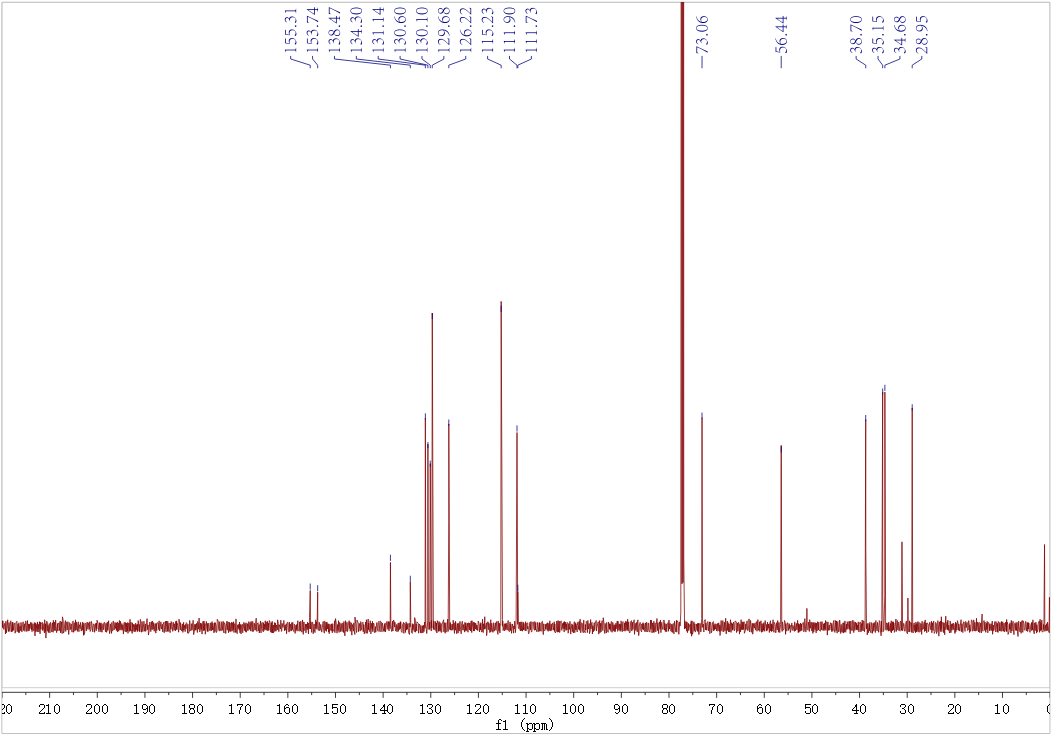


### Fig. S34. ^13^C NMR spectrum of compound 7-*E* (CDCl**_3_**, 150 MHz)


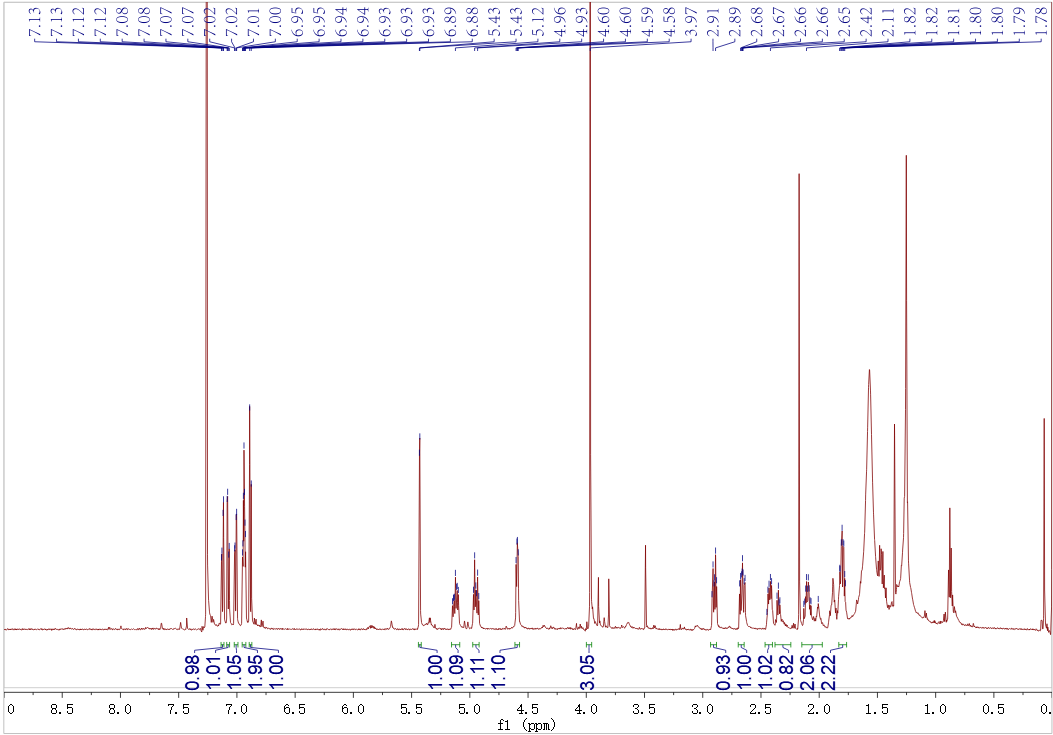


### **Fig. S35.** ^1^H NMR spectrum of compound **6-*E*** (CDCl_3_, 600 MHz)


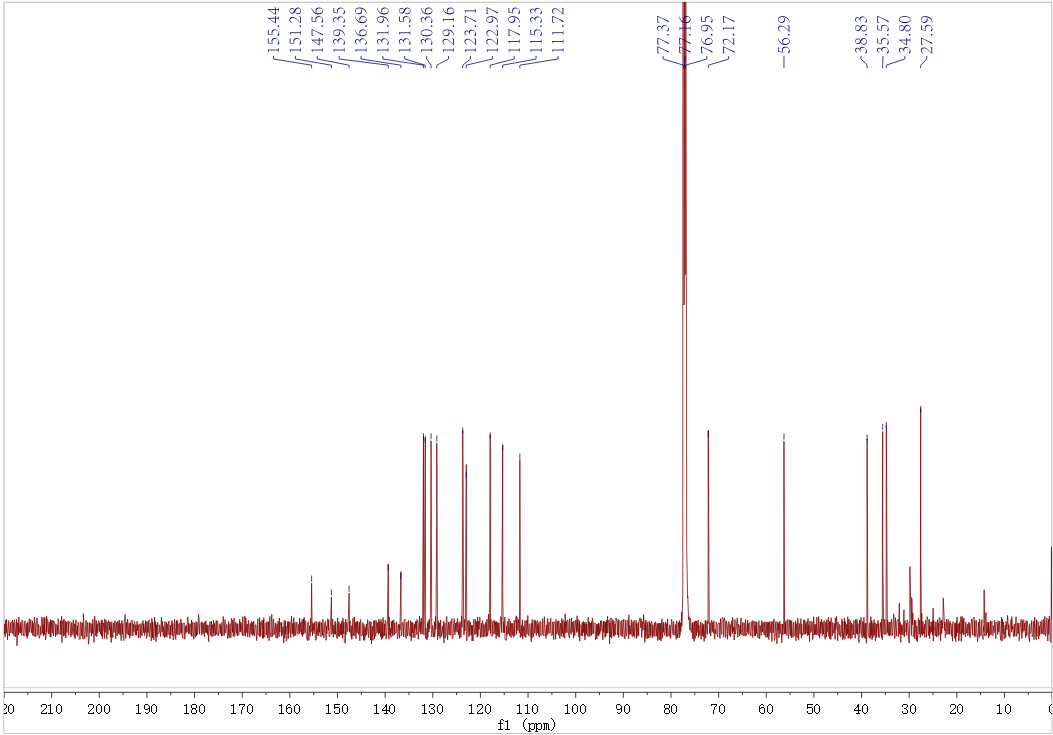


### Fig. S36. ^13^C NMR spectrum of compound 6-*E* (CDCl**_3_**, 150 MHz)


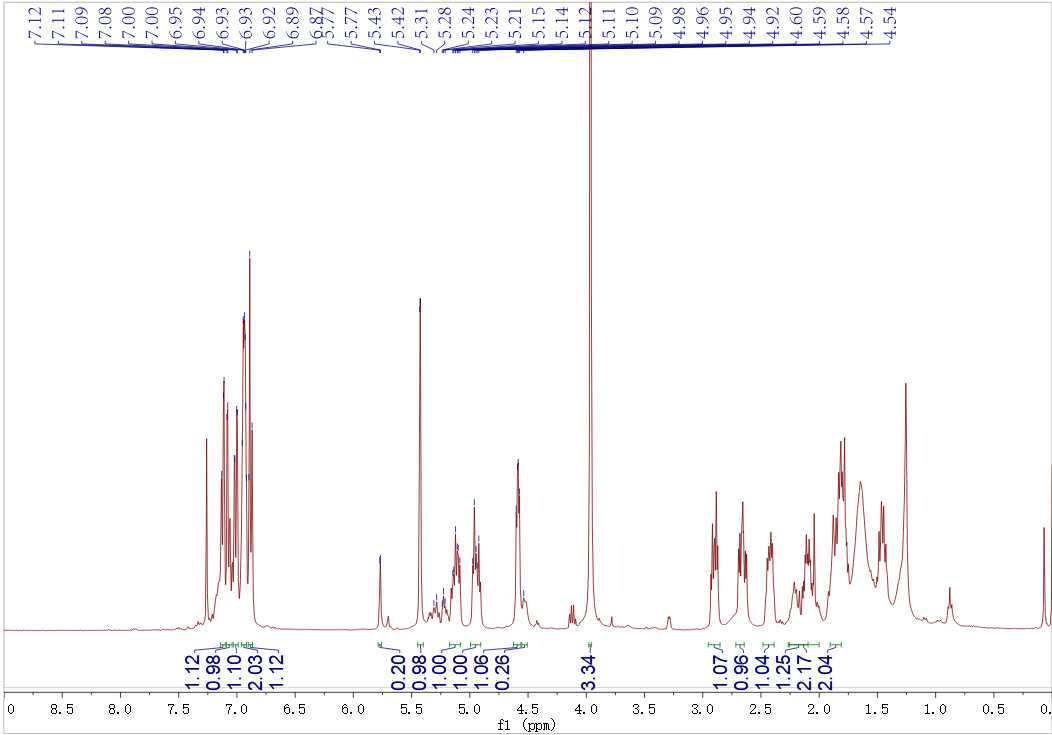


### Fig. S37. ^1^H NMR of compound 6 (provided to determine the ratio of *cis*/*trans* isomers, CDCl_3_, 600 MHz)


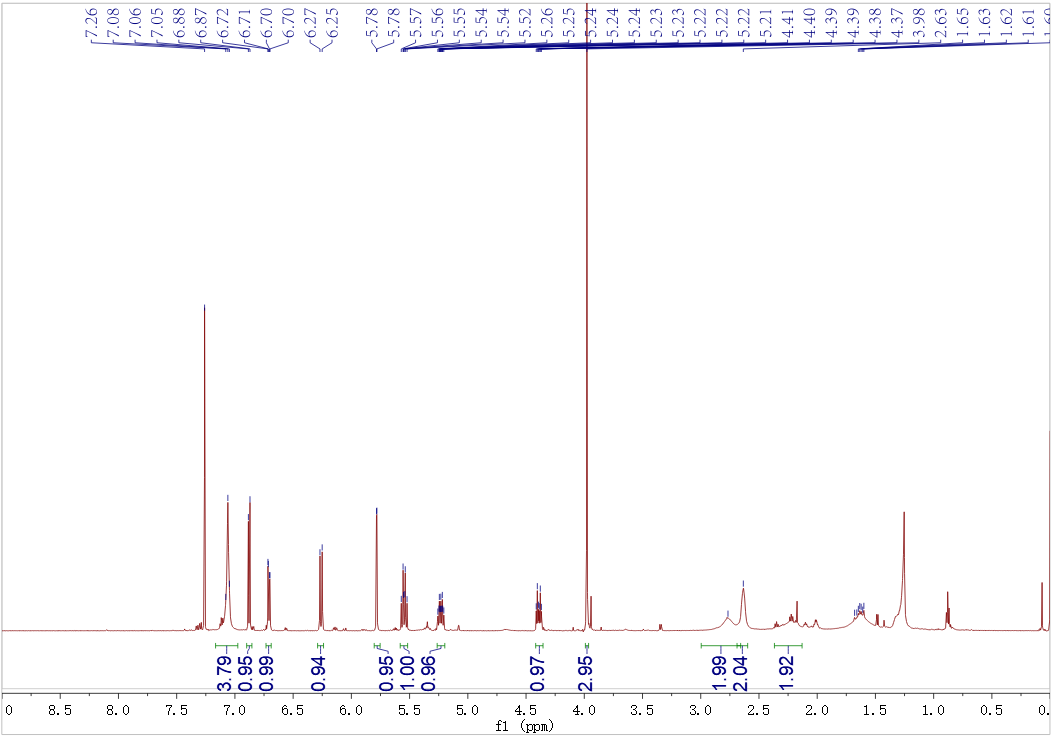


### **Fig. S38.** ^1^H NMR spectrum of compound **13** (CDCl_3_, 600 MHz)


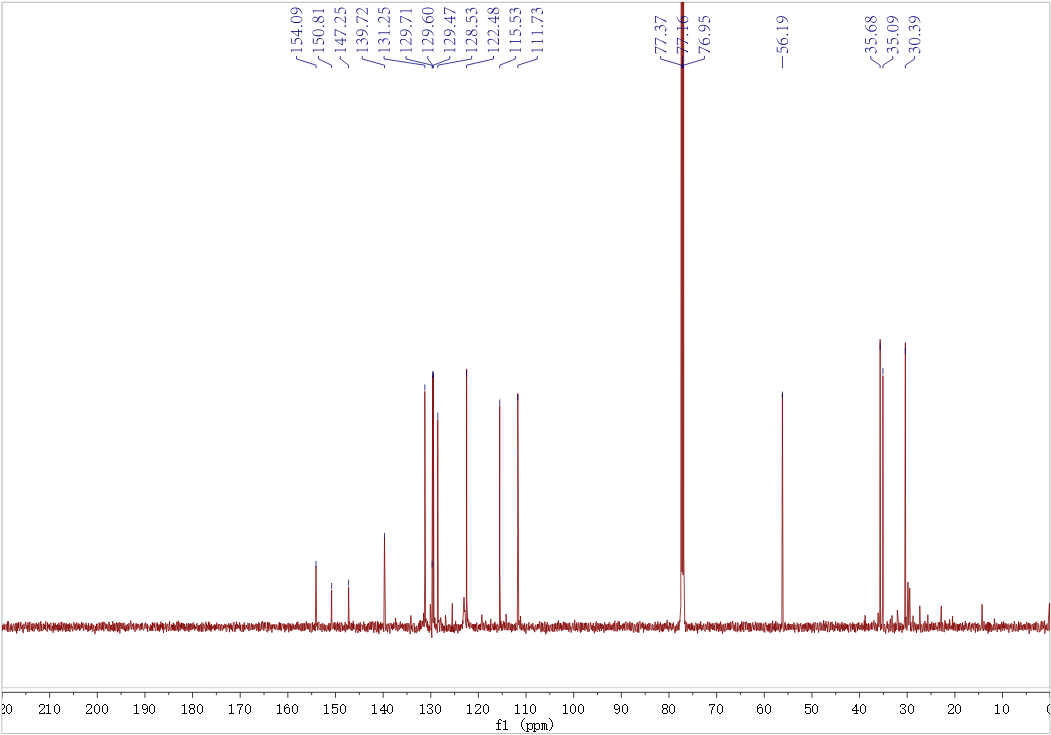


### Fig. S39. ^13^C NMR spectrum of compound 13 (CDCl**_3_**, 150 MHz)


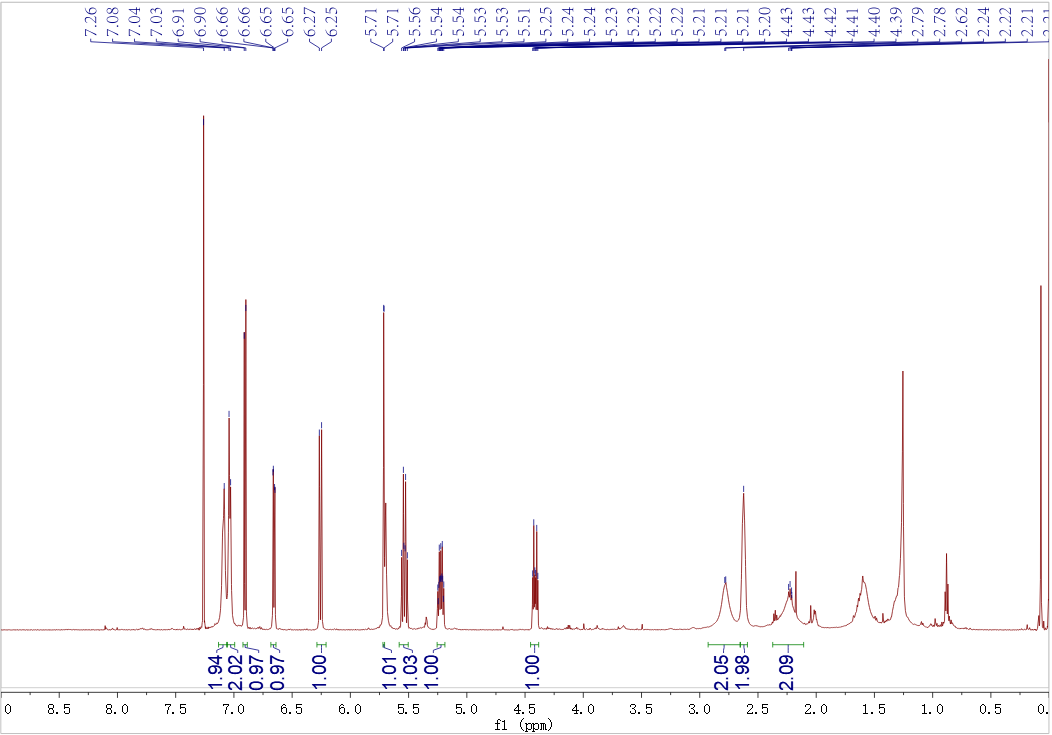


### **Fig. S40.** ^1^H NMR spectrum of compound **2** (CDCl_3_, 600 MHz)


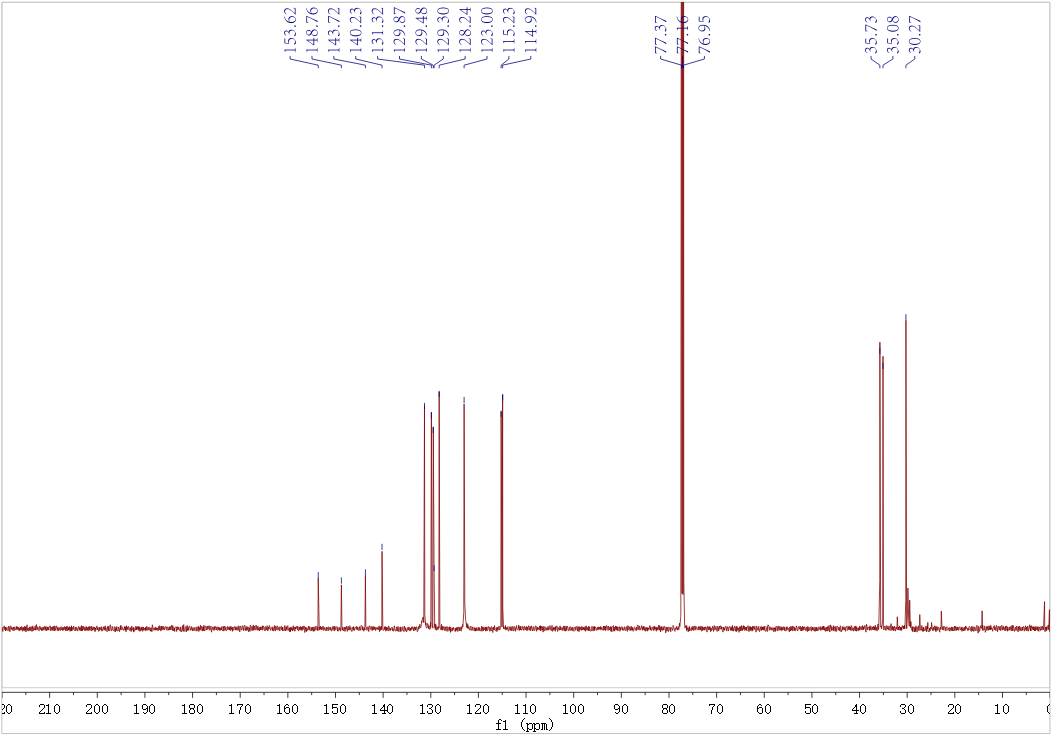


### Fig. S41. ^13^C NMR spectrum of compound 2 (CDCl**_3_**, 150 MHz)

## 7. HRESIMS spectrum data of important compounds


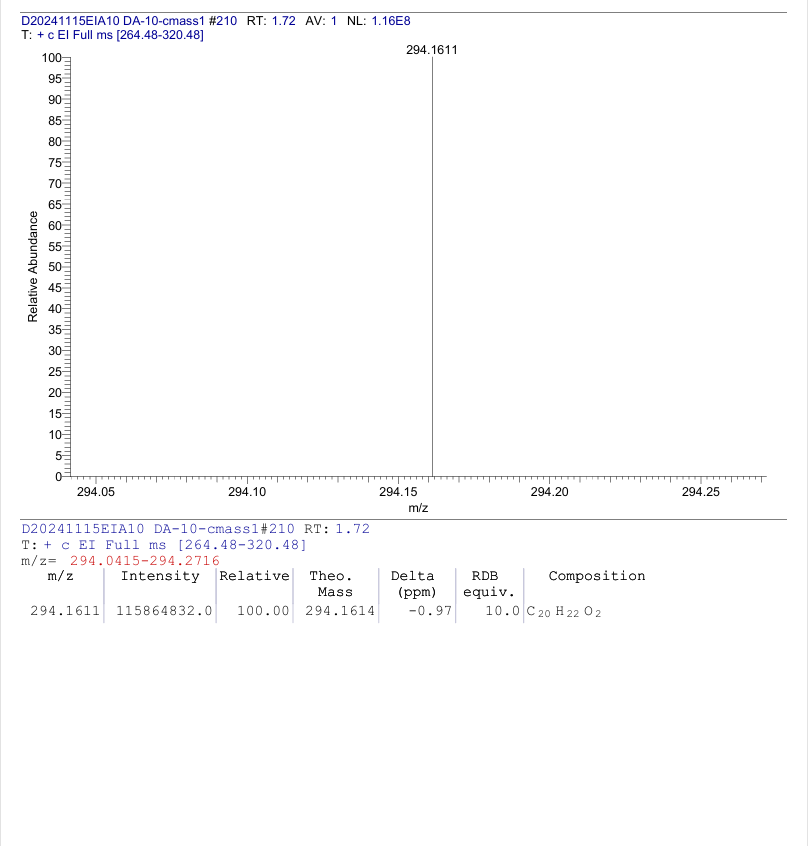


### Fig. S42. HRESIMS spectrum of otteacumiene O (1)


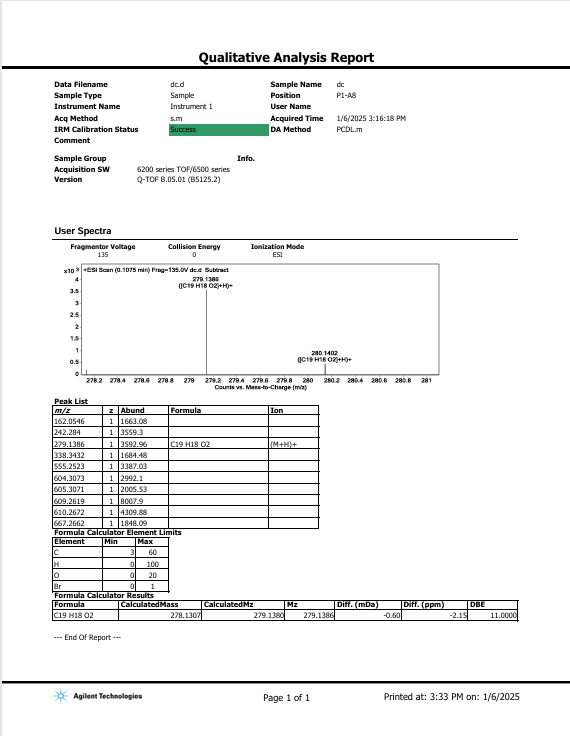


### **Fig. S43.** HRESIMS spectrum of otteacumiene P(**2**)

**
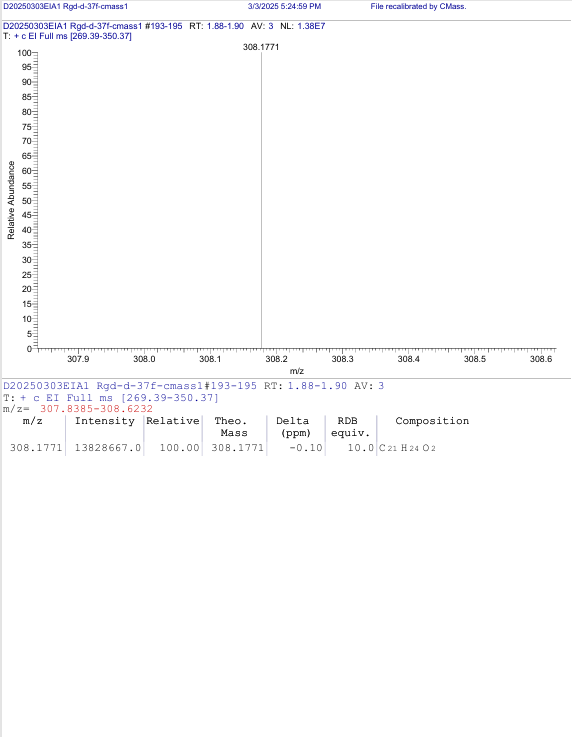
**

### Fig. S44. HRESIMS spectrum of compound 3

## 8. Comparison of NMR data of natural 1 and synthetic 1

| Comparison of NMR data between natural **1** and synthetic **1** | | | | | |
| --- | --- | --- | --- | --- | --- |
| No. | *δ*_C_ (natural **1**) | *δ*_H_ (*J* in Hz) | *δ*_C_ (synthetic **1**) | | *δ*_H_ (*J* in Hz) |
| 1 | 130.5 | 6.30, d (15.8) | 130.5 | 6.31, d (15.8) | |
| 2 | 127.3 | 6.08, dt (15.8, 6.6) | 127.4 | 6.09, dt (15.8,6.6) | |
| 3 | 36.5 | 2.84, m | 36.5 | 2.84, m | |
| 4 | 129.3 | 5.48, dt (15.3,5.9) | 129.3 | 5.48, dt (15.3,5.9) | |
| 5 | 131.6 | 5.54, dt (15.3,5.9) | 131.5 | 5.54, dt (15.3,5.9) | |
| 6 | 35.6 | 2.28, m  2.26, m | 35.6 | 2.28, m  2.26, m | |
| 7 | 35.7 | 2.58, m | 35.7 | 2.59, m | |
| 1’ | 131.3 |  | 131.3 |  | |
| 2’(6’) | 127.9 | 7.30, d (8.8) | 127.9 | 7.31, d (8.7) | |
| 3’(5’) | 114.7 | 6.85, d (8.8) | 114.7 | 6.86, d (8.7) | |
| 4’ | 159.8 |  | 159.8 |  | |
| 1’’ | 133.5 |  | 133.5 |  | |
| 2”(6”) | 130.1 | 7.02, d (8.5) | 130.2 | 7.02, d (8.5) | |
| 3”(5”) | 115.8 | 6.72, d (8.5) | 115.9 | 6.74, d (8.5) | |
| 4” | 156.3 |  | 156.3 |  | |
| 4”-OMe | 55.4 | 3.77, s | 55.5 | 3.78, s | |

**δ* in parts per million, *J* in Hz, and obtained at 600/150 MHz. ^b^The NMR solvent was acetone-*d*_6_.


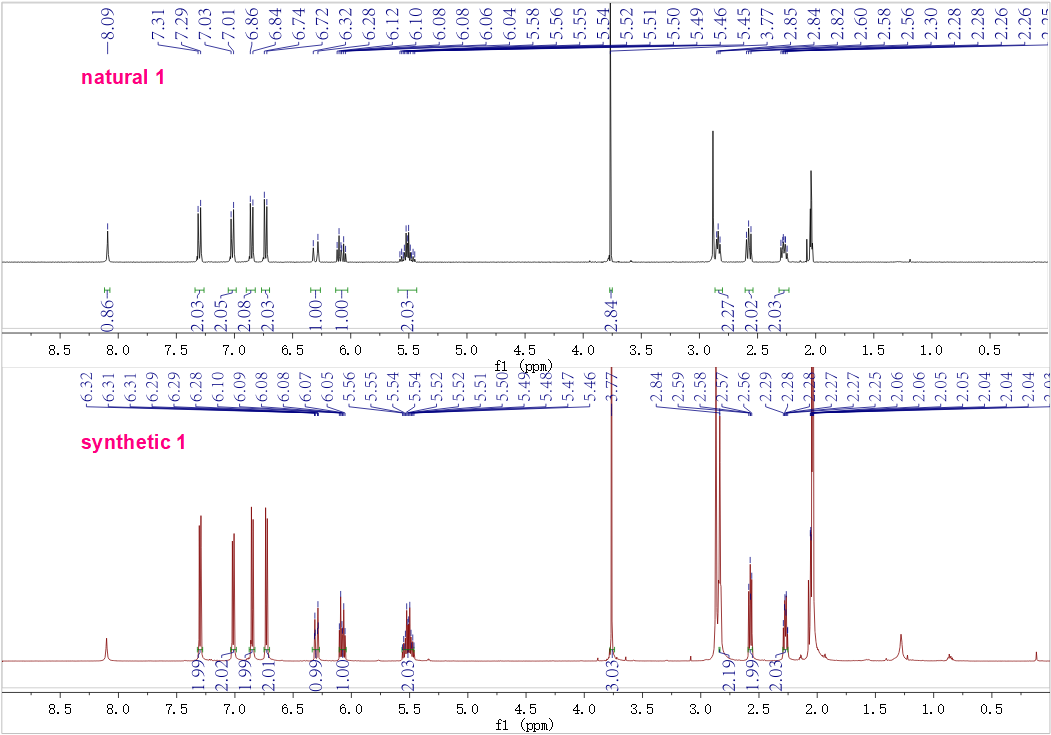


### Fig. S45 Comparison of ^1^H NMR data of natural 1 and synthetic 1


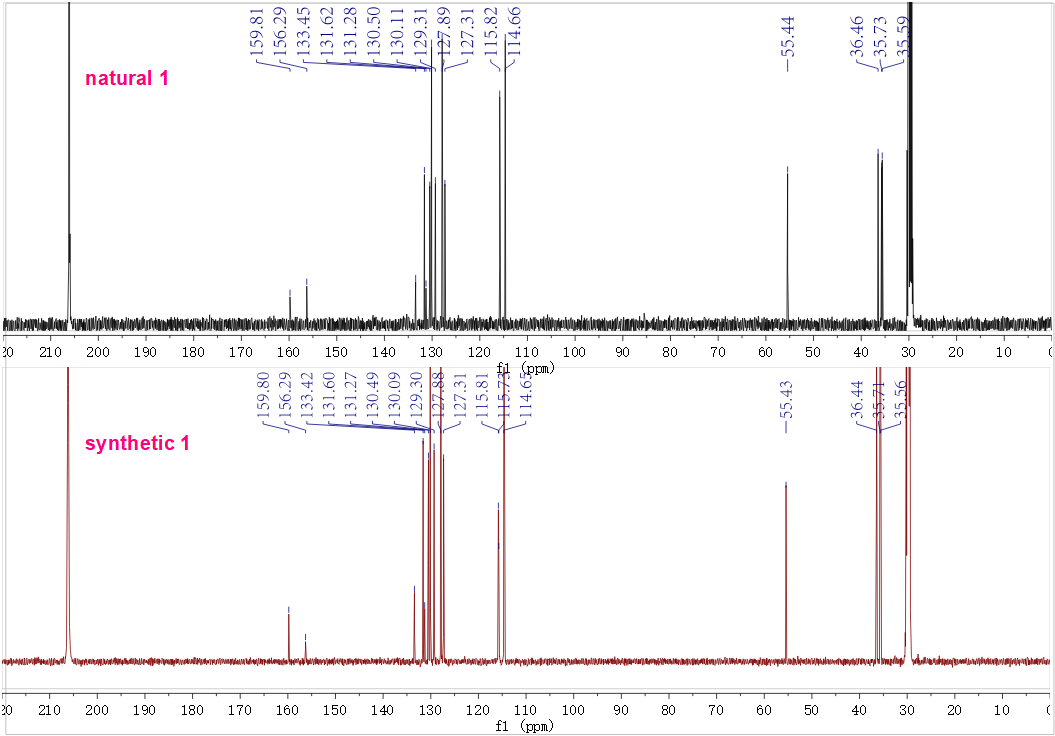


### Fig. S46 Comparison of ^13^C NMR data of natural 1 and synthetic 1

## 9. Comparison of NMR data of natural 3 and synthetic 3

| Comparison of ^13^C NMR data between natural **3** and synthetic **3** | | |
| --- | --- | --- |
| No. | *δ*_C_ (natural **3**) | *δ*_C_ (synthetic **3**) |
| 1 | 129.6 | 129.6 |
| 2 | 127.0 | 127.0 |
| 3 | 35.9 | 35.9 |
| 4 | 130.8 | 130.8 |
| 5 | 128.6 | 128.6 |
| 6 | 34.7 | 34.7 |
| 7 | 35.0 | 35.0 |
| 1’ | 130.6 | 130.8 |
| 2’ | 129.3 | 129.3 |
| 3’or3” | 113.9 | 113.9 |
| 3’or3” | 113.7 | 113.7 |
| 4’or4” | 158.7 | 158.7 |
| 4’or4” | 157.7 | 157.7 |
| 1’’ | 134.1 | 134.1 |
| 2” | 127.1 | 127.1 |
| 4’-OMe | 55.3 | 55.3 |
| 4”-OMe | 55.2 | 55.2 |

| Comparison of ^1^H NMR data between natural **3** and synthetic **3** | | | |
| --- | --- | --- | --- |
| No. | *δ*_H_ (*J* in Hz) (natural **3**) | | *δ*_H_ (*J* in Hz) (synthetic **3**) |
| 1 | 6.28, dt (15.5,1.7) | 6.28, dt (15.7,1.6) | |
| 2 | 6.05, dt (15.5,6.5) | 6.05, dt (15.8,6.6) | |
| 3 | 2.87, m | 2.87, m | |
| 4 | 5.50, dt (16,6) | 5.50, dt (16,6) | |
| 5 | 5.54, dt (16,6) | 5.53, dt (16,6) | |
| 6 | 2.30, m | 2.33, m | |
| 7 | 2.64, t (7.5) | 2.63, t (8.8) | |
| 2',6' | 7.27, d (8.5) | 7.27, d (8.6) | |
| 3',5' | 6.84, d (8.5) | 6.84, d (7.4) | |
| 2'',6'' | 7.10, d (8.5) | 7.10, d (8.6) | |
| 3'',5'' | 6.82, d (8.5) | 6.82, d (7.1) | |
| 4’-OMe | 3.80, s | 3.80, s | |
| 4”-OMe | 3.78, s | 3.80, s | |

**δ* in parts per million, *J* in Hz, and obtained at 600/150 MHz. ^b^The NMR solvent was CDCl_3_.

## 10. Comparison of NMR data of natural 2 and synthetic 2

| Comparison of NMR data between natural **2** and synthetic **2** | | | | | |
| --- | --- | --- | --- | --- | --- |
| No. | *δ*_C_(natural **2**) | *δ*_H_ (*J* in Hz) | *δ*_C_(synthetic **2**) | | *δ*_H_(*J* in Hz) |
| 1 | 143.7 |  | 143.7 |  | |
| 2 | 148.8 |  | 148.8 |  | |
| 3 | 114.9 | 5.71, d (2.1) | 114.9 | 5.71, d (2.1) | |
| 4 | 129.3 |  | 129.3 |  | |
| 5 | 123.0 | 7.03, d (8.0) | 123.0 | 7.04, d (8.0) | |
| 6 | 115.2 | 6.66, d (8.0) | 115.2 | .6.66, d (8.3) | |
| 7 | 129.9 | 6.26, d (11.5) | 129.9 | 6.26, d (11.5) | |
| 8 | 128.2 | 5.54, dt (11.5,9.1) | 128.2 | 5.54, dt (11.6,9.2) | |
| 9 | 30.3 | 2.63, t (7.5) | 30.3 | 2.62 s | |
| 10 | 131.3 | 4.41, dt (15.3,5.6) | 131.3 | 4.41, dt (15.345.6) | |
| 11 | 129.5 | 5.22, dt (15.3,7.8) | 129.5 | 5.22, dt (15.4,7.7) | |
| 12 | 35.7 | 2.24, m | 35.7 | 2.24, m | |
| 13 | 35.1 | 2.78, m | 35.1 | 2.79, m | |
| 1’ | 153.6 |  | 153.6 |  | |
| 2’(6’) |  |  |  |  | |
| 3’(5’) |  |  |  |  | |
| 4’ | 140.2 |  | 140.2 |  | |

**δ* in parts per million, *J* in Hz, and obtained at 600/150 MHz. ^b^The NMR solvent was CDCl_3_.


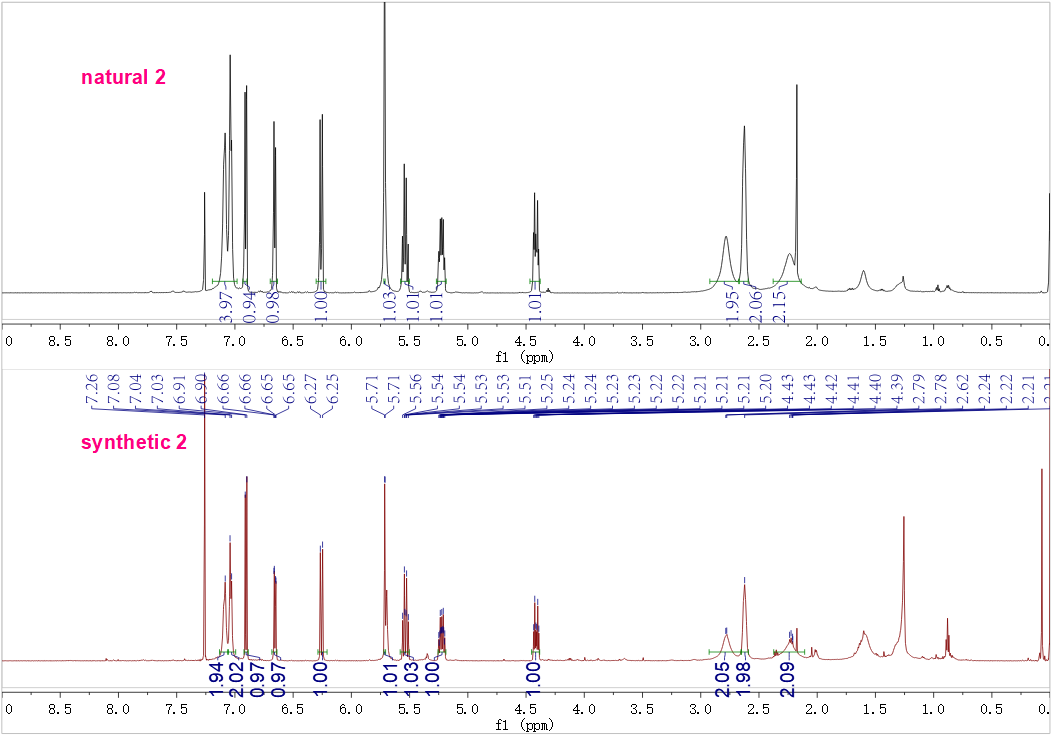


### Fig. S47. Comparison of ^1^H NMR data of natural 2 and synthetic 2


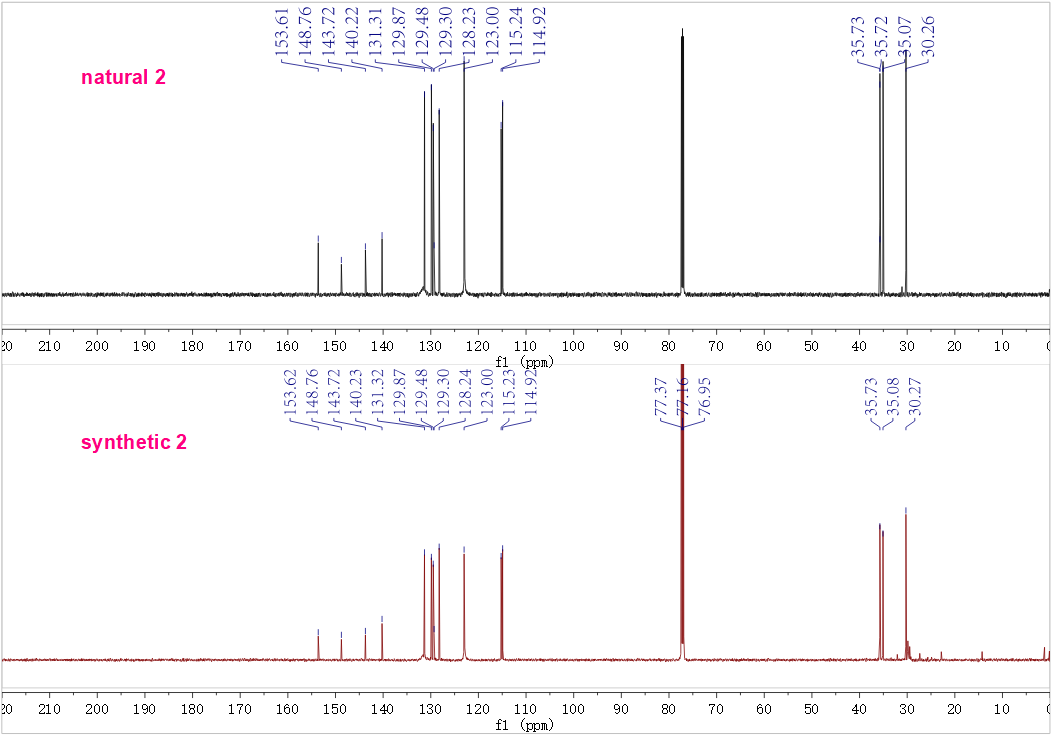


### Fig. S48. Comparison of ^13^C NMR data of natural 2 and synthetic 2

1. *Correspondence:

   Xing-Ren Li, E-mails: lixingren@mail.kib.ac.cn;

   Li-Dong Shao, E-mails: shaolidong@ynucm.edu.cn;

   Gang Xu, E-mails: Xugang008@mails.kib.ac.cn. [↑](#footnote-ref-1)
